# Reenacting Neuroectodermal Exposure of Hematopoietic Progenitors Enables Scalable Production of Cryopreservable iPSC-Derived Human Microglia

**DOI:** 10.1007/s12015-022-10433-w

**Published:** 2022-08-15

**Authors:** Mona Mathews, Jannis Wißfeld, Lea Jessica Flitsch, Anahita Shahraz, Vesselina Semkova, Yannik Breitkreuz, Harald Neumann, Oliver Brüstle

**Affiliations:** 1 LIFE & BRAIN GmbH, Venusberg-Campus 1, 53127 Bonn, Germany; 2 Institute of Reconstructive Neurobiology, University of Bonn Medical Faculty and University Hospital Bonn, Venusberg-Campus 1, 53127 Bonn, Germany; 3 Institute of Reconstructive Neurobiology, Neural Regeneration Group, University of Bonn Medical Faculty and University Hospital Bonn, Venusberg-Campus 1, 53127 Bonn, Germany

**Keywords:** Human microglia, Induced pluripotent stem cells, Bioreactor, Macrocarrier, In vitro differentiation

## Abstract

Human microglia, as innate immune cells of the central nervous system (CNS), play a central role in the pathogenesis of a large number of neurological and psychiatric disorders. However, experimental access to primary human microglia for biomedical applications such as disease modeling is extremely limited. While induced pluripotent stem cells (iPSCs) could provide an alternative source of microglia, the reenactment of their complex ontogenesis with a yolk sac origin and subsequent priming upon CNS invasion has remained a challenge. Here, we report a developmentally informed in vitro differentiation method for large-scale production and cryopreservation of iPSC-derived microglia (iPSdMiG). Specifically, iPSCs were propagated in conditions yielding both yolk sac hematopoietic derivatives and early neuroepithelial cells. To enable large-scale production, we implemented 3D bioreactor-based dynamic culture conditions and the use of novel mesh macrocarriers. Under these conditions, microglia could be harvested across a time period of at least 6 weeks, with 1 × 10^6^ iPSCs giving rise to up to 45 × 10^6^ iPSdMiG. The transcriptomic profile of iPSdMiG showed high similarity to adult human microglia, and harvested cells were immunopositive for typical microglial markers. In addition, iPSdMiG were able to secrete pro-inflammatory cytokines, engaged in phagocytotic activity, produced reactive oxygen species and lent themselves to co-culture studies in neural 2D and 3D systems. Importantly, iPSdMiG were efficiently cryopreserved, enabling the establishment of donor-specific microglia cell banks for disease modeling, drug discovery and eventually cell therapy.

**Graphical abstract:**

**Main points.**

Scalable generation of iPSC-derived multi-lineage embryoid bodies on macrocarriers, reproducibly releasing microglia exhibiting characteristic markers and function. Cells are transcriptomically similar to primary human microglia and cryopreservable.

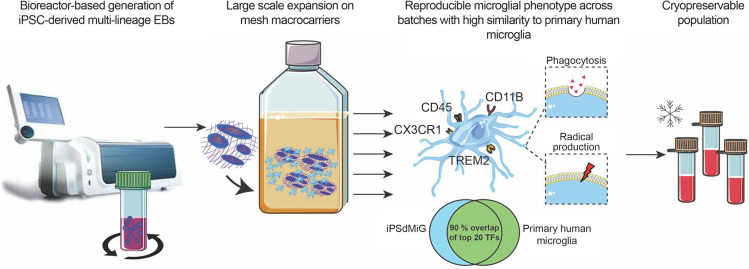

**Supplementary Information:**

The online version contains supplementary material available at 10.1007/s12015-022-10433-w.

## Background

Microglia, the innate immune cells of the central nervous system (CNS), have recently become a key focus of biomedical research in nervous system homeostasis and disease. On the one hand, microglia are implicated in physiological processes such as synaptic surveillance and pruning [1–3], phagocytosis of apoptotic cells [4], oligodendrocyte development and myelination [5–7], as well as neuromodulation [8]. On the other hand, they are involved in the pathogenesis of various CNS disorders by initiating, exacerbating and modulating the disease process [9, 10]. Genome-wide association and exome sequencing studies have revealed several risk variants for neurodegenerative diseases in genes expressed in microglia [11–13]. Thus, microglia play a dual role in CNS diseases: While they are responsible for detecting and resolving pathogenic insults, their uncontrolled activation might lead to chronic inflammation, tissue damage and aggravation of disease pathology [14, 15]. This contrasting role is further complicated by the highly dynamic nature of microglia, with regard to their activation state and secretory phenotype, which may constantly change during disease progression [14, 16, 17].

Experimental studies on human microglia and their implications in health and disease have been limited by the highly restricted access to primary brain-resident cell types. However, this has partially been bypassed by the advent of cell reprogramming and the generation of induced pluripotent stem cells (iPSCs). Today, a large number of in vitro cell programming and differentiation protocols enable the generation of disease-specific neural cell types, such as populations of neural stem cells, neuronal subtypes, astrocytes and oligodendrocytes, from patient-derived somatic cells such as skin fibroblasts and blood cells. The majority of these in vitro differentiation protocols aim at recapitulating developmental pathways relevant to cell type specification [18], and the pertinence of this approach has been impressively illustrated in the context of midbrain dopamine neuron generation. For this highly disease-relevant neuronal subtype, the in vitro recapitulation of its floor plate origin during iPSC differentiation significantly increased the authenticity of the in vitro-derived counterpart [19, 20].

Unlike other CNS-resident cell types, microglia are derived from the hematopoietic system [7]. Hematopoiesis occurs in waves, where early embryonic hematopoiesis has been shown to give rise to microglia [21], while peripheral myeloid cells such as blood monocytes and peripheral macrophages are generated at later stages [22–25]. Fate-mapping studies have more specifically shown that microglia originate from early erythromyeloid precursors in the yolk sac. These cells mature across multiple states into a cluster of differentiation (CD) 45 and CX3C chemokine receptor 1-double-positive immediate precursor population that invades the developing brain and gives rise to permanently CNS-resident mature microglia [21, 22, 24]. In recent years, several methods describing the generation of microglial precursors or microglial-like cells from iPSCs have been published. However, in these protocols, the cells typically lack exposure to a neural environment during differentiation, which is a critical step in native microglia development and maturation. The recapitulation of this stepwise maturation process and difficulties with the cryopreservation of iPSC-derived microglial precursors or microglial-like cells necessitate further optimization [26–31].

Here, we set out to replicate the sequential in vivo development of microglia in the yolk sac and embryonic CNS by using 3D culture conditions that expose emerging iPSC-derived erythromyeloid precursors to early neuroepithelial cells. Translation of this protocol to a bioreactor-compatible novel macrocarrier system with a subsequent cryopreservation step enables the generation of large numbers of functionally validated iPSC-derived microglia (iPSdMiG), which can be applied as an ‘off-the-shelf’ product for disease-related research, drug screening and other biomedical applications.

## Methods

### Induced Pluripotent Stem Cell Culture

IPSCs were cultured in StemMACS™ iPSbrew media (Milteyni Biotech, Bergisch Gladbach, Germany) on 6-well tissue culture plates coated with geltrex (180 μg/ml; Thermo Fischer Scientific, Waltham, Massachusetts). Medium changes were performed every other day and lines were replated twice per week using 0.5 mM ethylenediaminetetraacetic acid (EDTA; Sigma Aldrich, St. Louis, Missouri) in 1 × DPBS (Gibco, Thermo Fischer Scientific) for dissociation.

### In vitro Differentiation and Expansion of Human iPSdMiG

Formation of multi-lineage embryoid bodies (EBs) was induced from evenly sized iPSC colonies in adherent culture. 2D-cultured iPSC colonies were detached with collagenase (1 mg/ml; Gibco) at 37 °C for 30 min. The detached free-floating colonies were transferred into EB basal media composed of N2 supplement (1x; Gibco), B27 supplement (1x; Gibco) and small molecules as described below. EB suspension culture was carried out dynamically in a table top bioreactor (CERO 3D bioreactor, OLS, Bremen, Germany) with daily media changes as follows: EB basal media was supplemented with 10 ng/ml recombinant human (rh) bone morphogenetic protein 4 (BMP4; Peprotech, Thermo Fischer Scientific) on day 0, with 10 ng/ml rhBMP4 and 20 ng/ml rh fibroblast growth factor 2 (FGF2; R&D systems, Minneapolis, Minnesota) on day 1, and with 10 ng/ml rhBMP4, 10 ng/ml rhFGF2, 1 ng/ml rh activin A (ACT A; Peprotech) and 3 µM of a wingless/integrated (WNT)-pathway inhibitor (WNTC59; Xcess Biosciences, Chicago, Illinois) on day 2. Finally, media was changed to EB basal media without additional small molecules on day 3. On day 4, EBs were manually inoculated on macrocarriers.

The non-woven hydrophilic nylon mesh discs (sold as ‘nylon net filters’ from Merck, Darmstadt, Germany) employed as macrocarriers in this study (patent: WO2021180781A) were originally described for use as particle filtration membranes. They measure 47 mm in diameter, with hydrophilic nylon fibres fused to form a mesh structure of 60 μm pore size and a porosity of 41%. Before use, mesh discs were sterilized at 120 °C using a standard autoclave and coated with 5 μg/ml poly-L-ornithine (PLO; Sigma Aldrich) followed by 1 μg/ml fibronectin (Sigma Aldrich). Each coating step was performed for 24 h at 37 °C. Meshes were washed three times in sterile 1 × DPBS (Gibco) before and after each coating step.

Following inoculation, macrocarriers were first kept in static culture in a humidified incubator for up to 48 h in order to facilitate adherence and initial outgrowth of progenitors across the surface of the macrocarriers. Once firmly adhered, EB-loaded macrocarriers were transferred into suspension culture formats such as T75 non-tissue culture-coated flasks and placed on a rocker within a humidified incubator for further differentiation in expansion media composed of STEMdiff^TM^APEL^TM^2 (Stem Cell Technologies, Vancouver, Canada), 10% knock-out serum replacement (KSR; Gibco), 1 × N2 supplement, 1 × B27 supplement, 25 ng/ml rh interleukin (IL) 3 (rhIL3), 25 ng/ml rhIL34 and 25 ng/ml rh macrophage colony stimulating factor (rhMCSF; all from Peprotech).

IPSdMiG were harvested by collecting medium from suspension cultures, which was then passed through a 40 μm cell strainer (Thermo Fischer Scientific). Cells were pelleted by centrifugation at 400 × g for 5 min and seeded in adherent culture on poly-L-lysine (PLL)-coated wells (5 μg/ml; Sigma Aldrich). Seeding was carried out in medium consisting of DMEM/F12 with HEPES (Gibco), 1 × N2 supplement and 2 mM Glutamax (Gibco), supplemented with 100 ng/ml rhIL34 and 50 ng/ml rh transforming growth factor β (TGFβ; Peprotech). Twenty-four hours after seeding in monoculture, cells were used for experiments.

### Immunocytochemical Analyses

Cells on macrocarriers or in 2D culture were fixed with 4% paraformaldehyde (PFA; Alfa Aeser, Thermo Fischer Scientific) in 1 × DPBS for 10 min at room temperature. Subsequently, specimens were incubated for 1 h in blocking buffer consisting of 10% fetal calf serum (FCS; Gibco), which was supplemented with 0.1% Triton X-100 (Sigma Aldrich) for macrocarriers. Samples were incubated with primary antibodies overnight at 4 °C in respective blocking buffers. Primary antibodies included mouse anti-CD31 (1 μg/ml; Thermo Fischer Scientific), rabbit anti-tubulin beta III (TUBB3 antibody clone TUJ1; 1 μg/ml; Biolegend, Perkin Elmer, Waltham, Massachusetts), rabbit anti-ionized calcium-binding adapter molecule 1 (IBA1; 1 μg/ml; FujiFilm, Tokyo, Japan), rabbit anti-CX3C chemokine receptor 1 (2.5 μg/ml; ProSci Inc, Poway, California), rabbit anti-purinergic P2Y12 receptor (1:100; Merck) and rabbit anti-transmembrane protein 119 (TMEM119; 1:100; Abcam, Cambridge, UK). Following washing in 1 × DPBS, specimens were incubated with corresponding secondary antibodies Alexa Fluor 488 anti-mouse (1 μg/ml; Invitrogen, Thermo Fischer Scientific), Alexa Fluor 488 anti-rabbit (1 μg/ml; Thermo Fischer Scientific) or Alexa Fluor 555 anti-rabbit (1 μg/ml; Biolegend) for 1 h at room temperature in respective blocking buffers. Finally, nuclei were counterstained with 4’- 6’ diamidino-2-phenylindole (DAPI; 2 μg/ml; Sigma Aldrich) for 15 min at room temperature. Images were acquired with a Leica fluorescence microscope DMI 6000B and post-processed using Image J v1.52p image analysis software.

### Flow Cytometry

For pluripotency validation, expression of T cell receptor alpha locus-1–60 (TRA-1–60) on iPSCs was analyzed. IPSCs were dissociated by treatment with StemPro Accutase (Thermo Fischer Scientific), and detached cells were pelleted by centrifugation at 200 × g for 3 min. Cells were stained with TRA-1–60 primary antibody (2 μg/ml; Merck) for 1 h on ice, followed by secondary antibody Alexa Fluor 488 anti-mouse for 30 min on ice. Omission of the primary antibody was used to determine potential unspecific binding.

For determining the proliferative index of iPSdMiG, intranuclear staining with Ki67 (1:500; DCS, Hamburg, Germany) was assessed by flow cytometry. Both, suspension iPSdMiG harvested directly from differentiation flasks and adherent iPSdMiG collected 24 h after seeding (described below; see section ‘In vitro differentiation and expansion of human iPSdMiG’), were analyzed using the BD (Franklin Lakes, New Jersey) transcription factor (TF) buffer kit according to the manufacturer’s protocol.

For live staining of EBs, these were first dissociated into single cells by incubating with StemPro Accutase for 10 min at 37 °C. Dissociated EBs in single cell suspension were then stained with primary antibodies against anti-CD235a-APC (4 μg/ml; BD) together with anti-CD34-FITC (1:50; Milteyni Biotech), and anti-CD45-APC (1:20; BD) together with CX3C chemokine receptor 1-Alexa Fluor 488 (2 μg/ml; Santa Cruz, Dallas, Texas) for 1 h on ice.

Live staining of iPSdMiG surface markers was carried out by seeding cells at a density of 1 × 10^6^ cells per well in 6-well tissue culture plates using the seeding protocol described above (see section ‘In vitro differentiation and expansion of human iPSdMiG’). Twenty-four hours after seeding, cells were mechanically detached using a cell lifter and incubated with primary antibodies for 1 h, followed by secondary antibody incubation for 30 min on ice. The following primary antibodies were used to characterize iPSdMiG: rat anti-CD11B (2.5 μg/ml; BD), biotinylated-CD45 (1:20; BD), rabbit anti-CX3C chemokine receptor 1 (2.5 μg/ml; ProSci Inc) and mouse anti-triggering receptor expressed on myeloid cells 2 (TREM2; 2.5 μg/ml; R&D systems). Corresponding secondary antibodies used were Cy5 anti-rat (2 μg/ml; Jackson Immuno Research, Bar Habor, Maine), Alexa Fluor 488-streptavidin (2 μg/ml; Invitrogen), PE anti-rabbit (2 μg/ml; Jackson Immuno Research) and Alexa Fluor 488 anti-mouse (2 μg/ml).

All flow cytometry analyses were performed using a BD ACCURI™ C6 plus flow cytometer (BD) and analyzed using FlowJo 10.5.3 software (BD).

### Secretome Profiling

IPSdMiG were seeded in 96-well imaging plates (Ibidi, Graefelfing, Germany) as described above (see section ‘In vitro differentiation and expansion of human iPSdMiG’) at a density of 2 × 10^4^ cells per well in monoculture or at a density of 1.4 × 10^4^ cells per well in co-culture with 7 × 10^4^ long-term self-renewing neuroepithelial-like stem cell (lt-NES)-derived neurons per well (described below; see section “Co-culture of iPSdMiG with lt-NES derived cortical spheroids”). After 24 h, supernatants of iPSdMiG monocultures were collected for quantifying interferon γ (IFNγ), IL1β, IL6, IL8, IL10, IL12p70 and tumor necrosis factor α (TNFα) concentrations using the 7-plex human pro-inflammatory cytokine and chemokine kit (Meso Scale discovery, Rockville, Maryland) according to the manufacturer’s protocol. For co-cultures, cells were cultured for 5 days in neural media (described below; see section ‘Co-culture of iPSdMiG with lt-NES derived cortical spheroids’). Thereafter, wells were either unstimulated or stimulated with the pro-inflammatory stimuli lipopolysaccharide (LPS; 1 μg/ml, Invivogen, San Diego, California) or IFNγ (50 ng/ml; Sigma Aldrich), or with the anti-inflammatory factor IL4 (20 ng/ml; Sigma Aldrich) in neural media. After 24 h of stimulation, supernatants were collected for analysis using the 7-plex human pro-inflammatory cytokine and chemokine kit (Meso Scale discovery) and the MesoScale Discovery QuickPlex SQ120 system according to the manufacturer’s protocols.

### Phagocytosis and Reactive Oxygen Species Assays

Twenty-four hours after seeding iPSdMiG, wells were either treated solely with 50 μg pHrodo-red labeled *Staphylococcus aureus* bioparticles (*S. aureus*; Thermo Fischer Scientific) or co-stimulated with 1 μg/ml LPS for 1.5 h at 37 °C. Negative control samples were pre-treated for 1 h with 4 μM cytochalasin D (CytoD; Thermo Fischer Scientific), which inhibits actin polymerization and thereby prevents active phagocytosis of bioparticles by iPSdMiG [32]. After washing out residual bioparticles, three images were acquired per condition using a Leica fluorescence microscope, and analyzed with Image J v1.52p analysis software. The background intensity of each picture was subtracted before fluorescence intensities were normalized to the respective DAPI nuclei count for each picture. Finally, results were normalized to the untreated condition.

For the detection of reactive oxygen species (ROS) production associated with phagocytosis, iPSdMiG were seeded as described above (see section ‘In vitro differentiation and expansion of human iPSdMiG’), and ROS detection was carried out as previously described [33]. For negative control, iPSdMiG were pre-incubated with 4 mM N-acetyl-cysteine (NAC; Sigma Aldrich) for 1 h at 37 °C. Subsequently, cells were treated with 20 μg unlabeled *S. aureus* BioParticles (Thermo Fisher Scientific) for 15 min at 37 °C. After washing out residual bacterial particles, cells were treated with 30 μM dihydroethidium (DHE; Invitrogen) in Krebs-HEPES-buffer (Sigma Aldrich) for 15 min at 37 °C. Cells were then fixed with 4% PFA containing 0.25% glutaraldehyde (Sigma Aldrich) for 10 min at room temperature, washed and directly subjected to imaging. Using an INCell Analyzer 2200 system (GE Healthcare, Chicago, Illinois), nine images were acquired per condition and analyzed with Image J v1.52p. The background intensity was subtracted in each image, before the DHE intensity in each experimental condition was normalized to the respective DAPI nuclei count, and finally normalized to the untreated condition.

### RNA Sequencing, Differential Expression and Pathway Enrichment Analyses

For RNA sequencing, iPSdMiG were seeded for 24 h as described above (see section ‘In vitro differentiation and expansion of human iPSdMiG’) and total RNA was extracted by lysing cells in RLT-buffer (Qiagen, Hilden, Germany) supplemented with β-mercaptoethanol (1:100; Sigma Aldrich). Subsequently, RNA was isolated using the RNeasy kit (Qiagen) according to the manufacturer’s instructions. RNA concentrations were determined using a Nanodrop 2000 (Thermo Fischer Scientific) and diluted to a concentration of 100 ng/μl. RNA sequencing was performed at the Next Generation Sequencing (NGS) Core Facility of the University Hospital Bonn with 1–2 × 10^7^ single-end reads per sample on a HiSeq 2500 V4 (Illumina, San Diego, California). Raw data from primary human microglia, human cortical tissue and iMGL were retrieved from previously published data sets deposited in GEO (GSE99074: human microglia and cortex, GSE89189: iMGL). Quality and adapter trimming were performed with BBDuk from BBMap (v38.86), followed by read alignment to the homo sapiens reference genome hg38 (GRCh38) with the ensemble gene annotation version 97 using STAR (v2.7.3a, [34]) using standard parameters. Read count generation was performed using the feature Counts/Subread (v2.0.0, [35]) ignoring multimapping reads. Unwanted variance due to differences in library preparation and sequencing was removed using RUVSeq (v1.22.0, [36]) for R (v4.0.2, [37]) in RStudio (v1.3.1056, [38]). For TF enrichment analysis, the microglia core signature was extracted using DESeq2 (v1.28.1, [39]) and apeglm (v1.10.0, [40]). Both, primary human microglia and iPSdMiG, were individually compared to iPSCs, and each data set was filtered for their specific microglia core signature with log_2_FC ≥ 3 and FDR-adjusted p-value ≤ 0.001. These highly microglial-specific genes were then used to calculate TF enrichment using ChE3A [41] and plotted with visNetwork (v2.0.9, [42]). Furthermore, a direct comparison of iPSdMiG to primary human microglia was performed, and expression changes (|log_2_FC|≥ 1 and FDR-adjusted p-value ≤ 0.01) were identified by pathway enrichment analysis using clusterProfiler (v3.16.1, [43]) and reactomePA (v1.32.0, [44]). Finally, weighted gene correlation network analysis was performed to identify co-expression of genes within the different cell types using weighted gene correlation network analysis (WGCNA; v1.69, [45]) for R and pathway enrichment for identified modules of interest were again realized with clusterProfiler and ReactomePA.

### Cryopreservation and Retrieval of iPSdMiG

IPSdMiG collected from cell culture medium were passed through a 40 μm cell strainer and either directly cryopreserved or pre-treated for 24 h with the anti-apoptotic molecule muristerone (5 μM; Biotechne). For cryopreservation, iPSdMiG were counted, pelleted by centrifugation at 400 × g for 5 min, resuspended in freezing medium containing 90% KSR and 10% dimethyl sulfoxide (DMSO; Sigma Aldrich) and immediately transferred to cryovials, which were stored in freezing boxes (Mr. Frosty™; Thermo Fischer Scientific) at -80 °C for 48 h and then at -150 °C for long-term storage. In parallel, the conditioned medium (*i.e.*, the cell-free supernatant after centrifugation) was stored at -80 °C for use during subsequent retrieval.

For thawing, cryovials stored at -150 °C were directly placed in a 37 °C water bath for 1 min. The thawed cell suspension was then quickly transferred to a falcon tube and diluted with 10 ml DMEM/F12 basal medium. Cells were pelleted by centrifugation at 400 × g for 5 min and resuspended in 3 ml of the respective conditioned media that was cryopreserved during cell freezing. This cell suspension was further diluted 1:1 with fresh STEMdiff^TM^APEL2 supplemented with 10% KSR, 1 × N2 supplement, 1 × B27 supplement, 25 ng/ml rhIL3, 25 ng/ml rhIL34 and 25 ng/ml rhMCSF. For muristerone pre-treated samples, 5 μM muristerone was also added as post-thaw treatment. Thawed iPSdMiG were cultivated in a non-tissue culture plate for 48 h at 37 °C and then seeded for subsequent experiments as described above (see section ‘In vitro differentiation and expansion of human iPSdMiG’). Phenotypic and functional analyses of cryopreserved cells were performed after 3 to 12 months of cryopreservation at -150 °C.

### Co-Culture of iPSdMiG with lt-NES Derived Neurons And Cortical Spheroids

An lt-NES cell line originally generated from the human embryonic stem cell line H9.2 according to a described protocol [46] was matured for 2 weeks in neural differentiation medium comprising a 1:1 mixture of DMEM/F12 basal medium supplemented with 1 × N2 and Neurobasal medium (Thermo Fischer Scientific) supplemented with 0.5 × B27 on geltrex-coated (1:50 dilution) tissue culture plates. Medium was changed every 2 days. Differentiated cells were detached using StemPro Accutase and cryopreserved. Upon thawing, these cultures were propagated in neural medium supplemented with ROCK inhibitor Y-27632 (10 μM; Hiss Diagnostics, Freiburg, Germany), BDNF (10 ng/ml; HiSS Diagnostics) and GDNF (10 ng/ml; HiSS Diagnostics). Specifically, 7 × 10^4^ cells were plated per well of 96-well imaging plates coated with geltrex (1:50 dilution) and further matured for 3 weeks at 37 °C and 6% CO_2_ in neural medium supplemented with BDNF and GDNF only. After neuronal maturation, iPSdMiG harvested from the medium of a running differentiation (iLB-C133bm-S4 line) were directly seeded onto the lt-NES cell-derived neuronal cultures at a density of 1.4 × 10^4^ iPSdMiG per well in neural medium supplemented with 100 ng/ml rhIL34. This co-culture was kept for a total of 6 days, with medium changes being performed every other day. On the 5^th^ day of co-culture, rhIL34 was withdrawn and wells were either left unstimulated or stimulated with the pro-inflammatory stimuli LPS (1 μg/ml) or IFNγ (50 ng/ml), or with the anti-inflammatory factor IL4 (20 ng/ml). After 24 h, the co-culture supernatant was collected for secretome analysis, and cells were fixed for immunocytochemical analysis.

For the generation of 3D cortical spheroids, a modified protocol from Pasca and colleagues [47] was used. In brief, iPSCs were incubated with Accutase for 8 min at 37 °C. Subsequently, 1.5 × 10^6^ single iPSCs were aggregated per AggreWell 800 well (Stem Cell Technologies) in spheroid differentiation medium (50% DMEM-F12 Glutamax (Gibco), 50% Neurobasal, 0.5 × N2, 0.5 × B27, 0.5 × MEM-NEAA (Thermo Fischer Scientific), 1 mM L-glutamine (Thermo Fischer Scientific), 10 μg/ml insulin (Sigma Aldrich) and 1:1000 β-mercaptoethanol) supplemented with two SMAD pathway inhibitors – dorsomorphin (1 μM; Sigma Aldrich) and SB-431542 (10 μM; AxonMedChem, Hanzeplein, Netherlands) – and ROCK inhibitor Y-27632 (10 μM). Spheroids were cultivated at 37 °C and 5% CO_2_. Medium was changed daily, while ROCK inhibitor Y-27632 was removed after the first 24 h. After 5 days, spheroids were dislodged from the AggreWell, transferred into a CERO tube (OLS) and placed into a rotating CERO table-top bioreactor. From day 5 to day 12, spheroid differentiation medium was changed every other day. On day 12, medium was changed to spheroid differentiation medium supplemented with rhFGF2 (10 ng/ml; Biotechne) for four days. From day 16 on, spheroids were maintained in spheroid maturation medium, *i.e.*, spheroid differentiation media without supplementation of dorsomorphin, SB-431542 and rhFGF2. At day 30 of cortical spheroid culture, 5 × 10^6^ iPSdMiG were added to a CERO tube containing 10 spheroids and placed back into the CERO bioreactor for 1 to 4 weeks of suspension co-culture in spheroid maturation medium supplemented with 100 ng/ml rhIL34. During the first 3 days of co-culture, intermittent dynamic agitation was carried out to facilitate the adherence of microglia to the spheroids and consequential migration. From day 3 onwards, the co-culture was carried out with continuous dynamic agitation. Spheroids containing iPSdMiG were fixed with 4% PFA for 10 min at room temperature, followed by immersion in 30% sucrose (Sigma Aldrich) overnight at 4 °C. Subsequently, spheroids were transferred into a cryomold, embedded with gelatine-sucrose solution (10% sucrose and 7.5% gelatine (Sigma Aldrich) in 1 × DPBS), shock-frozen in an ice bath of propanol and dry ice and subsequently stored at -80 °C. For immunofluorescence staining, 20 μm thick sections were prepared using a cryostat (Microm HM 560, Thermo Fischer Scientific). Immunocytochemical analysis was performed as described above (see section ‘Immunocytochemical analyses’).

### Statistical Analyses

Data from time course analysis of surface marker expression and protein secretion across harvests were tested for normal distribution using Shapiro–Wilk test and a linear regression fit of r^2^ > 0.7. If these assumptions were met, Pearson’s correlation test was carried out; if not, Spearman’s correlation test was performed. All other data were tested for the assumptions of linear models, *i.e.*, normal distribution and homogeneity of variances, using the Shapiro–Wilk and Brown-Forsythe tests, respectively. If these assumptions were met, parametric paired or unpaired Student’s t-test, one-way ANOVA or two-way ANOVA with Bonferroni post-hoc tests were performed as indicated. If these assumptions were not met, non-parametric equivalents such as the Welch’s t-test or Kruskal–Wallis test with Dunn’s correction were performed. Data are depicted as mean ± standard deviation in the case of n < 3 and as mean ± standard error of the mean (SEM) in the case of n ≥ 3. The acronym n.s indicates ‘not significant’, * p < 0.05, ** p < 0.01 and *** p < 0.001. Statistical analyses were performed using GraphPad Prism version 8.3.1 (GraphPad software, Inc., San Diego, California).

## Results

### Implementation of a Multistep Differentiation Paradigm Enables the Scalable Differentiation of iPSdMiG

**Fig. 1:**
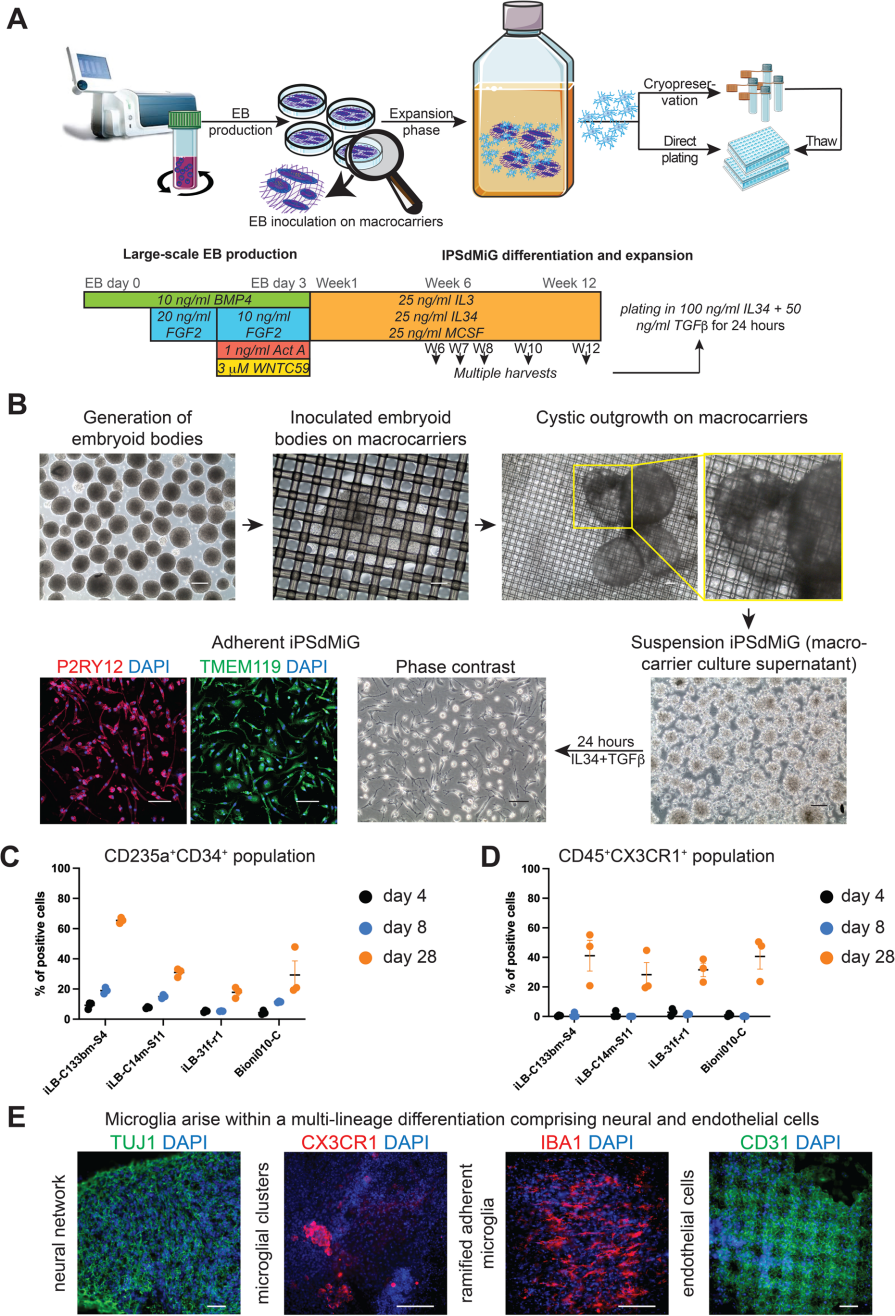
Developmentally informed multi-lineage in vitro differentiation of iPSdMiG. **A** Schematic illustration depicting the generation of iPSdMiG via scalable production of EBs that are fated to exhibit both primitive hematopoiesis and early neural precursors, and are subsequently propagated on mesh macrocarriers. Microglia can be harvested continuously from week 6 up to week 12 of differentiation. Upon propagation in 2D culture for 24 h, cells are assay-ready. Alternatively, suspension cells can be cryopreserved directly after harvesting. Abbreviation: W = week. **B** Phase contrast images showing suspension EBs (picture captured on day 4 of differentiation), which are inoculated on macrocarriers. Images captured on day 6 after seeding suspension EBs show outgrowth on macrocarriers, which remained adherent and continued expanding across the macrocarriers. Cystic structures are observed at week 6 of differentiation and round clusters of phase-bright suspension iPSdMiG are released into the medium of the macrocarrier culture by week 6, too. Harvested iPSdMiG that were seeded on tissue culture plates became adherent and ramified 24 h after seeding. Immunocytochemical analysis performed 24 h after seeding confirms the expression of TMEM119 (green) and P2Y12 receptor (red). Scale bars = 100 μm. **C** A hemogenic endothelial population was identified by the co-expression of CD235a and CD34 as early as day 4 of differentiation using flow cytometry analysis. n = 3 independent experiments, mean ± SEM. **D** A population of cells co-expressing CD45 and CX3C chemokine receptor 1 was identified at day 28 of differentiation using flow cytometry analysis. n = 3 independent experiments, mean ± SEM. **E** Immunocytochemical analysis of macrocarriers at week 12 of differentiation demonstrates that the multi-lineage differentiation culture successfully yielded TUBB3-positive cells (green) in dense neural networks, CX3C chemokine receptor 1-positive microglial clusters (red) and IBA1-positive ramified single microglial cells (red), as well as tile-like layers of CD31-positive endothelial cells (green). Scale bars = 100 μm

To simulate the hemogenic origin of microglial precursors and their subsequent maturation upon CNS invasion, iPSCs were allowed to form EBs containing early hematopoietic as well as neuroepithelial cells. To that end, EB development was directed by using rhBMP4 together with rhFGF2, rhACT A and WNTC59 in a step-wise manner (Fig. 1A). In total, EBs generated from four quality-controlled iPSC lines (3 male donors and 1 female donor) were subjected to microglia differentiation (Suppl. Fig. S1A). Four days after aggregation, EBs were inoculated in non-tissue culture plates containing nylon meshes coated with PLO and fibronectin, serving as macrocarriers, and further propagated in STEMdiff^TM^APEL^TM^2 medium supplemented with KSR, N2, B27, rhIL34, rhIL3 and rhMCSF (Fig. 1A, B).

On day 6, up to four disc-shaped meshes with a diameter of 47 mm and a pore size of 60 μm, each carrying inoculated EBs, were transferred into a single T75 suspension flask. Since EBs grew out on both sides of each macrocarrier, this provided a total surface area of up to 140 cm^2^ per T75 flask. EBs attached to the macrocarriers continued to increase in size and formed an expanding outgrowth spreading across the mesh. After 6 weeks of differentiation, this EB outgrowth had formed cysts (Fig. 1B), which further expanded into large macroscopic structures adhering to the macrocarriers up until week 12 of differentiation (Suppl. Fig. S2A). Phase contrast microscopy revealed that these cysts were densly filled with round phase-bright cells (Suppl. Fig. S2B). Immunofluorescence analysis of cryosections comprising both cystic and solid parts of the 12-week-old EBs further showed that the solid structures contained TUBB3-positive neurons, while the cystic structures were lined by CD31-positive cells (Suppl. Fig. S2C).

Starting from week 4 to week 6 of differentiation, small-sized (about 25 μm in diameter), phase-bright iPSdMiG were released from the differentiating culture into the medium, where they formed free-floating clusters of cells (Fig. 1B). Once harvested from the medium, iPSdMiG were seeded on PLL-coated tissue culture plates in medium containing rhIL34 and TGFβ. Twenty-four hours after seeding, adherent iPSdMiG displayed a ramified morphology and stained positive for the microglial markers P2Y12 receptor and TMEM119 (Fig. 1B). At this stage, the cells were subjected to further phenotypic and functional assays. In total, each of the four iPSC lines used in this study was differentiated at least four times in independent experiments. The number of harvested iPSdMiG ranged from 5 × 10^6^ to 45 × 10^6^ per million iPSCs, mostly depending on the genetic background. Whilst the iPSC lines iLB-C133bm-S4 and iLB-C14m-S11 were high-yielders in the range of 30 × 10^6^ to 45 × 10^6^ iPSdMiG per million iPSCs, iLB-31f-r1 provided a relatively moderate yield of 20 × 10^6^ to 30 × 10^6^ iPSdMiG per million iPSCs, whereas Bioni010-C iPSCs gave rise to only 5 × 10^6^ to 15 × 10^6^ iPSdMiG per million iPSCs.

### Evidence for Coinciding Hemogenic and Neuroepithelial Differentiation During Early iPSC Differentiation

Immunofluorescence and flow cytometry analyses were conducted to assess concomitant development of hemogenic and neuroepithelial differentiation in the maturing EBs. Starting within the first week after initiation of EB formation, flow cytometry revealed the emergence of a population of cells co-expressing CD235a and CD34 (Fig. 1C), a marker combination characteristic for primitive hematopoiesis [48]. By 4 weeks after EB induction, this fraction represented between 17.8% and 65.4% of the total population, depending on the individual iPSC line used. The proportion of cells co-expressing CD45 and CX3C chemokine receptor 1, a combination typically found in more mature microglial precursors that finally invade the developing brain [24], had increased from < 3% at days 4 and 8 to 28.3% to 41.1% at week 4 after EB induction, depending on the genetic background (Fig. 1D).

We further employed immunofluorescence analysis to assess neuronal, microglial and endothelial marker expression. Immunocytochemical analysis of day 4 EBs confirmed the presence of nestin- and sex determining region Y-box 2 (SOX2)-positive cells at this early stage of differentiation (data not shown). Twelve-week-old cultures contained a prominent population of neurons with TUBB3-positive neurites (Fig. 1E). At the same time, clusters of rounded as well as dispersed ramified microglial cells could be identified on the basis of their CX3C chemokine receptor 1 and IBA1 expression, respectively. In addition, numerous CD31-positive cells were found as a layer lining the macrocarriers in a tile-like manner (Fig. 1E). Taken together, these data indicate that the cell culture paradigm employed here promotes the emergence of microglial-like cells in a 3D culture setting supporting both hemogenic and neuroepithelial differentiation.

### Robustness of the in vitro Differentiation Process and Phenotypic Stability of iPSdMiG Across Time and Genotypes

Constant release of iPSdMiG into the media of macrocarrier cultures provides an attractive scenario for repetitive harvesting, provided that the generated cells exhibit sufficient phenotypic stability over time. To address this aspect, we assessed (i) surface protein expression, (ii) cytokine and chemokine secretion profiles as well as (iii) phagocytosis and (iv) ROS production across different harvesting time points (weeks 6, 7, 8, 10 and 12 of differentiation). These experiments were performed 24 h after plating harvested cells on PLL-coated dishes, using 3 genetically distinct iPSC lines (iLB-C133bm-S4, iLB-C14m-S11 and iLB-31f-r1), which were differentiated at least four times each in independent experiments.

#### Stable Surface Marker Expression Across Multiple Harvests

First, we compared freshly harvested iPSdMiG in suspension to adherent cultures generated by plating cells harvested at week 6 for 24 h. Using flow cytometry, we assessed the expression of the surface epitopes CD11B, CD45, CX3C chemokine receptor 1 and TREM2, and the proliferation marker Ki67(Suppl. Fig. S3A and B). Notably, for all cell lines tested, there were if at all only slight differences in surface marker expression between conditions. In addition, the expression levels of these markers were mostly similar in genetically distinct lines, except for TREM2 expression that varied across genotypes ranging from 10.7% to 26.2% in directly harvested cells (Suppl. Fig. S3A). Furthermore, we found no significant difference in the number of Ki67-positive cells between freshly harvested suspension iPSdMiG and adherent cultures (Suppl. Fig. S3B).

**Fig. 2:**
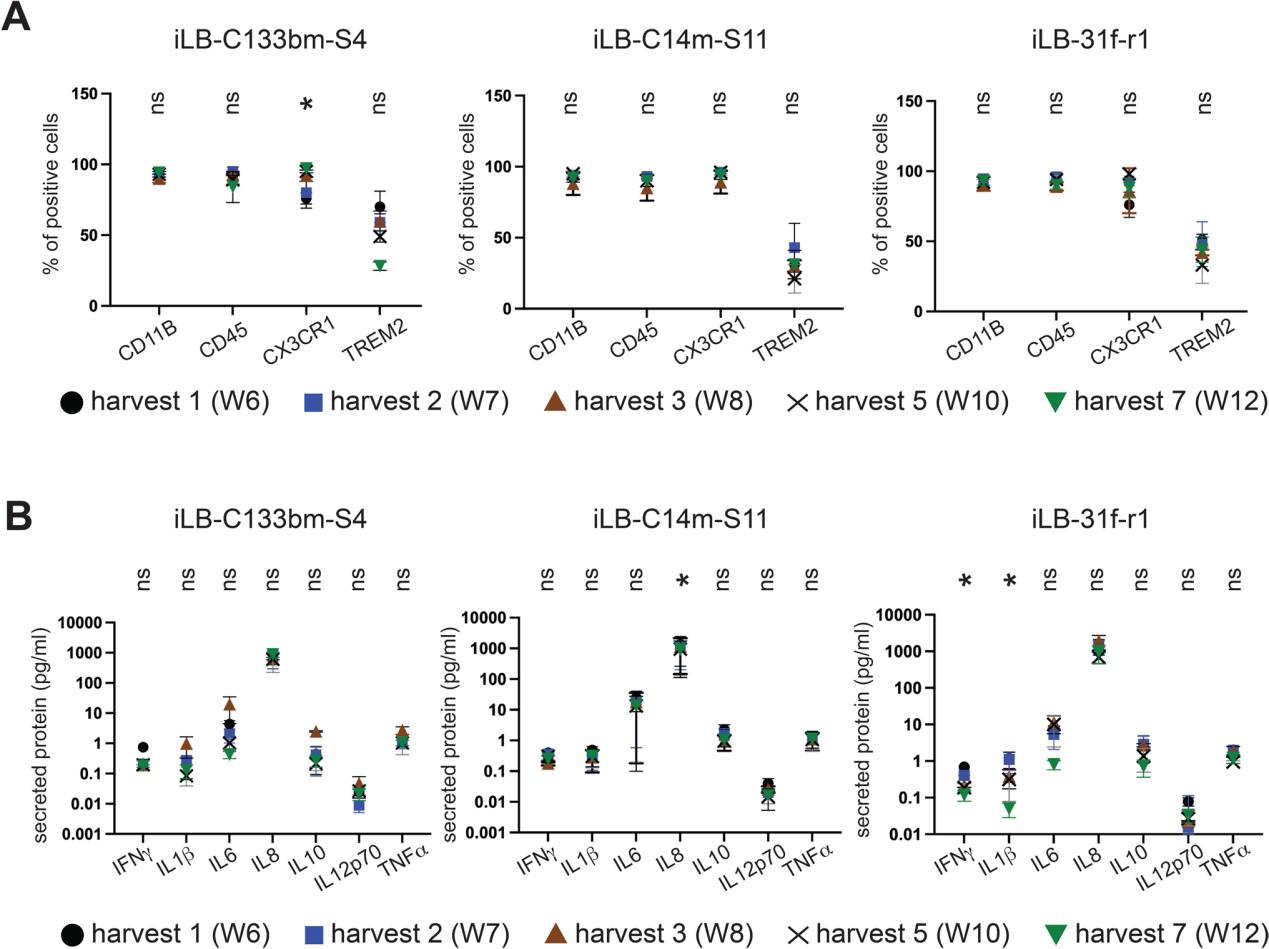
Surface marker expression and secretory phenotype of iPSdMiG remains stable across different harvesting time points and independent differentiation runs. **A** Flow cytometry analysis of surface marker expression showed comparable expression of the microglial markers CD11B, CD45, CX3C chemokine receptor 1 and TREM2 over time. n = 3 independent experiments, mean ± SEM. Data did not meet the criteria for normal distribution using Shapiro–Wilk test and/or linear regression fit of r^2^ > 0.7, and as such, Spearman’s correlation test was carried out for all samples. Significant correlation with time was observed for iLB-C133bm-S4 iPSdMiG in CX3C chemokine receptor 1 (p = 0.02; ρ = 1.0). * p < 0.05 as determined by correlation analysis. **B** Secretome profiles, analyzed using the Meso Scale Discovery human 7-plex pro-inflammatory kit, demonstrating predominantly consistent, albeit low level constitutive production of the inflammatory proteins IFNγ, IL1β, IL6, IL8, IL10, IL12p70 and TNFα. n = 3 independent experiments, mean ± SEM. Data did not meet the criteria for normal distribution using Shapiro–Wilk test and/or a linear regression fit of r^2^ > 0.7, and as such, Spearman’s correlation test was performed for all samples. Significant correlation with differentiation time was observed for IL8 secretion (p = 0.02; ρ = -1.0) of iLB-C14m-S11 iPSdMiG and for IFNγ (p = 0.02; ρ = -1.0) and IL1β (p = 0.02; ρ = -1.0) production of iLB-31f-r1 iPSdMiG. * p < 0.05 as determined by correlation analysis

We next assessed the expression of these surface markers across the 6-week interval of iPSdMiG harvesting. The results of this analysis confirmed stable expression of all markers over time for the cell lines iLB-C14m-S11 and iLB-31f-r1. Only iPSdMiG generated from iLB-C133bm-S4 showed a statistically significant positive correlation of CX3C chemokine receptor 1 expression and time of differentiation. Again, the overall percentages of cells positive for the individual markers were remarkably similar in the genetically distinct cell lines (Fig. 2A and Suppl. Table S1).

While iPSdMiG were typically harvested from week 6 to week 12, we noted that some iPSC lines such as iLB-C133bm-S4 and iLB-C14m-S11 produced harvestable suspension iPSdMiG already after 4 weeks of differentiation. We assessed microglial surface marker expression in these early week 4 harvests and found that expression levels of CD45, CX3C chemokine receptor 1 and TREM2 were similar to those observed in cells harvested between weeks 6 and 12 for both lines. However, CD11B was expressed in only about 80% and 74% of the iPSdMiG harvested at week 4 from iLB-C133bm-S4 and iLB-C14m-S11, respectively, representing a slightly lower fraction as compared to the more than 90% CD11B-positive iPSdMiG contained in harvests collected from week 6 onwards (Suppl. Fig. S3C).

#### Stable Constitutive Secretion of Inflammatory Proteins

We further analyzed constitutive secretion of the inflammatory cytokines and chemokines IFNγ, IL1β, IL6, IL8, IL10, IL12p70 and TNFα across the 6-week harvest period using a Meso Scale Discovery biomarker assay (Fig. 2B and Suppl. Table S2). Constitutive secretion of these 7 cytokines and chemokines was found to be mostly consistent across the different harvesting time points and genotypes. However, a statistically significant negative correlation of time in differentiation and IL8 secretion was found for iLB-C14-S11 iPSdMiG, as well as for IFNγ and IL1β production in iLB-31f-r1 iPSdMiG (Fig. 2B and Suppl. Table S2). Overall, the concentrations of the constitutively secreted inflammatory proteins were very low and partially even below the detection threshold of the assay (Suppl. Table S2). Taken together, these data show that iPSdMiG of different genetic backgrounds display a rather stable constitutive inflammatory cytokine release across the 6-week-harvesting time window, and the generally low secretion levels further point to an unactivated state of the immune cells generated.

### IPSdMiG Mount a Typical Inflammatory Immune Response to Activating Stimuli

**Fig. 3:**
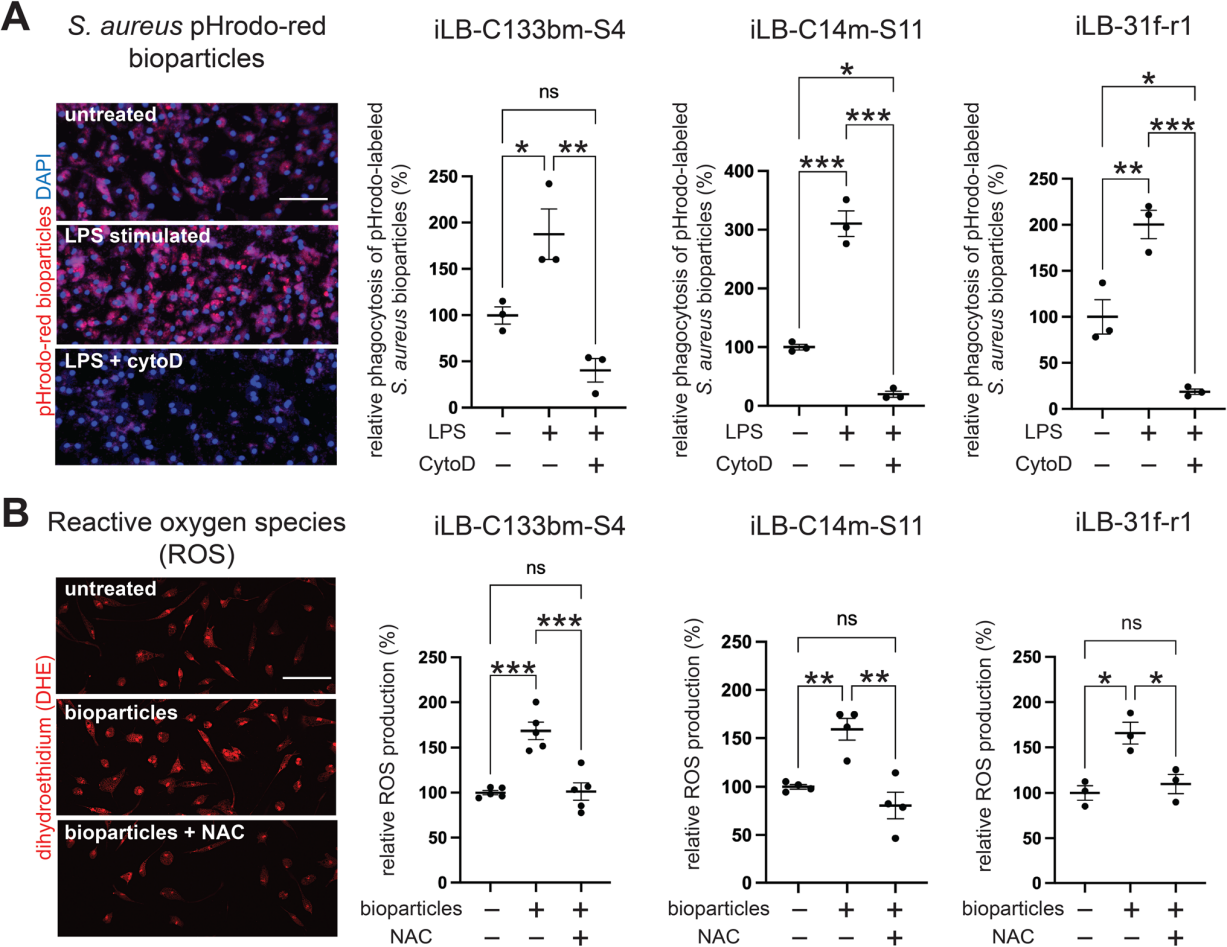
IPSdMiG respond to activation stimuli. **A** Representative fluorescence images (left; red = pHrodo fluorescence and blue = DAPI nuclei staining) and quantified data (right) showing that constitutive phagocytosis of pHrodo-labeled *S. aureus* bioparticles by iPSdMiG was increased by pro-inflammatory stimulation with LPS, and completely abolished after co-treatment with the actin polymerization destabilizer CytoD. n = 3 independent experiments, mean ± SEM. * p < 0.05, ** p < 0.01, *** p < 0.001 as determined by one-way ANOVA followed by Bonferroni post hoc test. Scale bar = 100 μm. **B** Representative fluorescence images (left; red = DHE fluorescence) and quantified data (right) depicting that constitutive production of ROS in iPSdMiG was increased by stimulation with *S. aureus* bioparticles and reduced again by concomitant treatment with the ROS scavenger NAC. n = 3–5 independent experiments, mean ± SEM. * p < 0.05, ** p < 0.01, *** p < 0.001 as determined by one-way ANOVA followed by Bonferroni post hoc test. Scale bar = 100 μm

A key characteristic of microglia is their ability to detect and phagocytose pathogenic material. Phagocytosis of bacterial particles induces oxidative bursts resulting in the production of ROS (as reviewed by [49, 50]). To assess the phagocytic capacity of iPSdMiG, cells were treated with pHrodo-tagged *S. aureus* bioparticles that emit a pH-dependent fluorescence signal in the acidic lysosomal compartment when phagocytosed. Constitutive phagocytosis was reproducibly observed in all iPSdMiG lines and could be further enhanced by treatment with LPS, which are capsule components of gram-negative bacteria acting as a pro-inflammatory activation signal (Fig. 3A). Specifically, LPS treatment resulted in a statistically significant 1.8- to 3.1-fold increase of constitutive baseline phagocytosis in different genetic backgrounds, whilst co-treatment with the mycotoxin CytoD, which blocks actin polymerization, completely abolished phagocytosis in LPS-stimulated iPSdMiG.

Next, we assessed phagocytosis-associated ROS production using DHE staining. To ensure comparability between the different conditions, cells were fixed after 15 min of DHE exposure [33]. Incubation of different iPSdMiG lines with *S. aureus* bioparticles significantly increased ROS production by 1.6- to 1.7-fold. ROS production was reduced to the level of unstimulated iPSdMiG by co-treatment of bioparticle-exposed cells with the antioxidant NAC (Fig. 3B). Overall, reproducible phagocytosis and phagocytosis-associated ROS production were observed in iPSdMiG from different genetic backgrounds and diverse harvesting time points ranging from week 6 up to week 12 of differentiation, further corroborating the stability of the microglial phenotype generated by our differentiation paradigm.

### IPSdMiG Closely Resemble ex vivo Primary Human Microglia On the Transcriptome Level

**Fig. 4:**
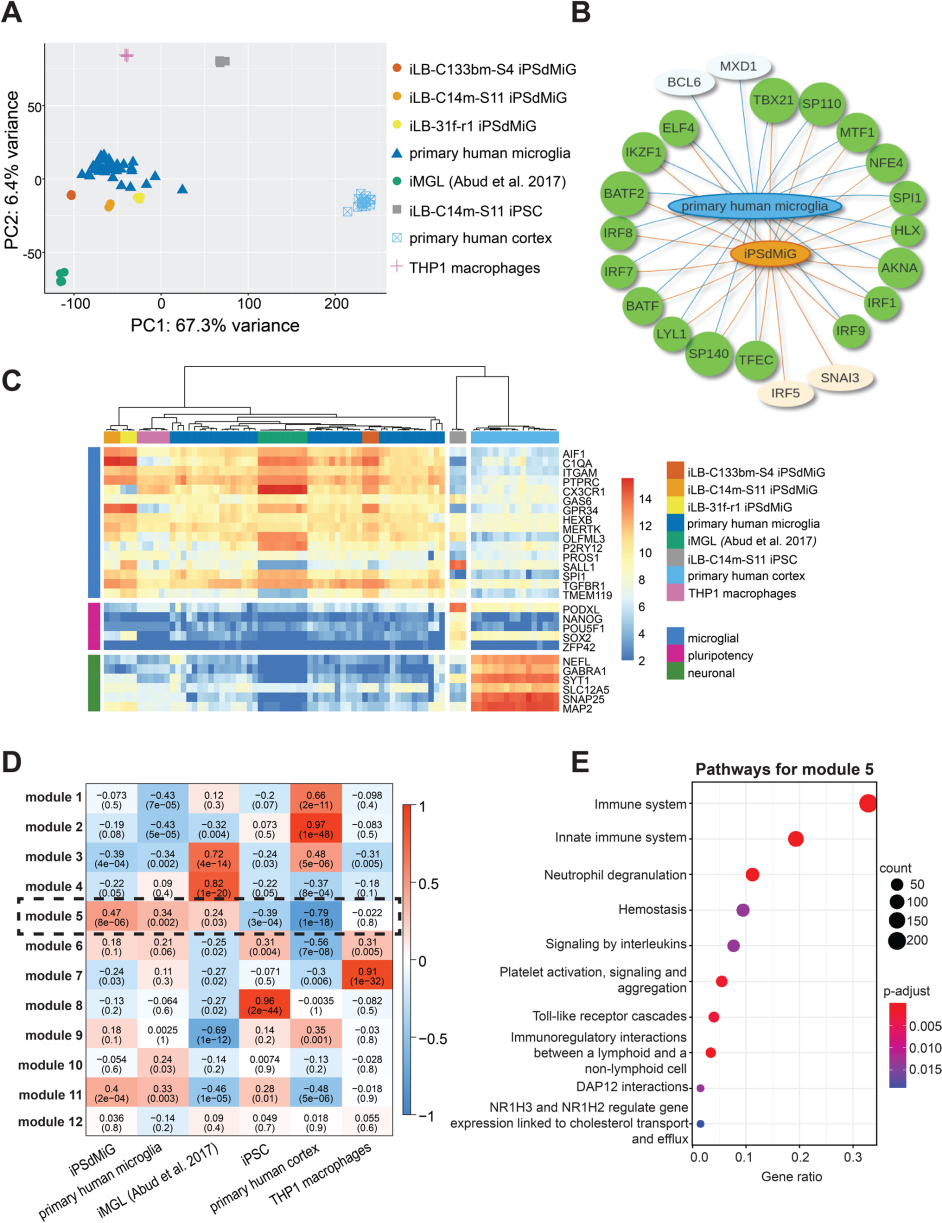
RNA sequencing analysis reveals close transcriptional similarity between primary human microglia and iPSdMiG. **A** PCA showing that iPSdMiG from all three lines (with n = 3 technical replicates per line) cluster in closest proximity to primary human microglia (n = 38 independent samples; in silico data) and comparatively farther away from iMGL (n = 9 independent samples; in silico data), THP1 macrophages (n = 6 technical replicates), iPSCs (n = 3 technical replicates) and cortex samples (n = 16 independent samples; in silico data). **B** TF enrichment analysis based on the gene core signature of iPSdMiG and primary human microglia (|log_2_FC|≥ 3 and FDR-adjusted p-value ≤ 0.001) identified a highly similar set of TFs driving cellular identity in both cell populations. Green circled genes were common in both primary human microglia and iPSdMiG. Pale orange circled genes were upregulated in iPSdMiG, while pale blue circled genes were upregulated in primary human microglia. **C** Heatmap of lineage-specific genes (microglial, pluripotency and neuronal genes) demonstrating that little to no pluripotency and neuronal gene transcripts are detectable in iPSdMiG and primary human microglia, whereas microglial genes are highly expressed in both cell types. **D** WGCNA identified a subset of genes (module five; dotted-black line) that were co-expressed in iPSdMiG, primary human microglia and iMGL. **E** Pathway enrichment analysis showed that module five primarily consisted of genes associated with innate immune pathways

Recent studies highlight that neural cues are important to maintain homeostatic expression of microglial markers and thus preserve microglial identity [51–53]. To investigate whether generation and maturation of iPSdMiG in our neural cell-enriched differentiation culture environment benefits microglial identity, we performed RNA sequencing studies and compared the transcriptome of our iPSdMiG to published RNA sequencing data from freshly isolated ex vivo primary human microglia [52] without subsequent in vitro culturing, THP1 macrophages and another iPSC-derived microglial-like population (iMGL, [28]). Principal component (PC) analysis (PCA) revealed that our three iPSdMiG lines (iLB-C133bm-S4, iLB-C14m-S11 and iLB-31f-r1) clustered in close proximity to primary human microglia and comparably farther away from other cell populations including THP1 macrophages (Fig. 4A). PC1 accounted for 67.3% of the variance and separated the different microglia and macrophage populations from iPSCs and more prominently from the included cortex samples. PC2, accounting for only 6.4% of the observed variance, separated the microglia samples from macrophages and iPSCs. After removal of all non-microglial cell types, which were responsible for the majority of variance observed in the PCA provided in Fig. 4A, iPSdMiG still clustered rather equidistantly to primary human microglia as compared to iMGLs (Suppl. Fig. S4A, left).

Next, differential expression analysis on core TF signatures of microglial identity was performed. To that end, respective core signatures were extracted by filtering for genes that are highly expressed in iPSdMiG and primary human microglia as compared to iPSCs (log_2_FC ≥ 3 and FDR-adjusted p value ≤ 0.001). Core signatures were then compared between both microglial cell types. The top 20 mean rank-enriched TFs were determined by ChEA3 [41], yielding a 90% overlap between iPSdMiG and primary human microglia. The TFs identified with high confidence were not only very similar for both core signatures, but also consisted of key genes driving microglial cell identity such as *SPI1* (coding for PU.1) and *interferon regulatory factor 8* (*IRF8)* ([24]; Fig. 4B), emphasizing the accuracy of the method as well as the microglial identity of iPSdMiG. To further substantiate this finding, gene sets characteristic for microglia, pluripotent stem cells and neurons were analyzed for their expression in our sample collection ([54–56]; Fig. 4C). IPSdMiG, iMGL and primary human microglia exhibited moderate to high expression of characteristic microglial genes (*complement C1q subcomponent subunit A (C1QA), CX3C chemokine receptor 1 (CX3CR1), P2Y12 receptor (P2RY12), SPI1* and *TMEM119*, among others) and low to no expression of pluripotent stem cell genes (*podocalyxin like (PODXL*; encoding for TRA-1–60), *NANOG*, *POU domain class 5 homeobox 1* (*POU5F1*; encoding for OCT4), *SOX2* and *zinc finger protein 42 (ZFP42*)), as well as neuronal marker genes (*neurofilament light polypeptide (NEFL)*, *gamma-aminobutyric acid receptor subunit alpha-1 (GABRA1)*, *synaptotagmin 1 (SYT1)*, *potassium-chloride transporter member 5* (*SLC12A5)*, *synaptosomal-associated protein 25 (SNAP25)* and *microtubule associated protein (MAP2))*. Focusing only on the microglial samples, hierarchical clustering based on the top 200 differentially expressed genes (DEGs) was carried out, further corroborating a high similarity between iPSdMiG and primary human microglia (Suppl. Fig. S4A, right).

WGCNA identified module 5 as particularly interesting for further analysis, since it specifically correlated with all three microglial samples (iPSdMiG, iMGL and primary human microglia; Fig. 4D; black dotted box). Pathway analysis on the genes in this module showed enrichment in innate immune pathways (Fig. 4E). Conversely, modules 2 and 8 were highly correlated with the cortex and iPSC samples, and were enriched in neuronal (Suppl. Fig. S4B) and stem cell-related pathways (Suppl. Fig. S4C), respectively. All other modules were mainly enriched for pathways related to metabolism, protein biosynthesis and/or RNA processing (data not shown).

Lastly, we set out to directly compare iPSdMiG to primary human microglia and analyzed expression changes by pathway enrichment analysis, which was dominated by differences in protein biosynthesis pathways, but not immune-related pathways (Suppl. Fig. S4D). Taken together, our RNA sequencing data corroborate the close transcriptomic proximity of our iPSdMiG to ex vivo primary human microglia (freshly isolated without subsequent in vitro culturing).

### IPSdMiG can be Efficiently Cryopreserved

**Fig. 5:**
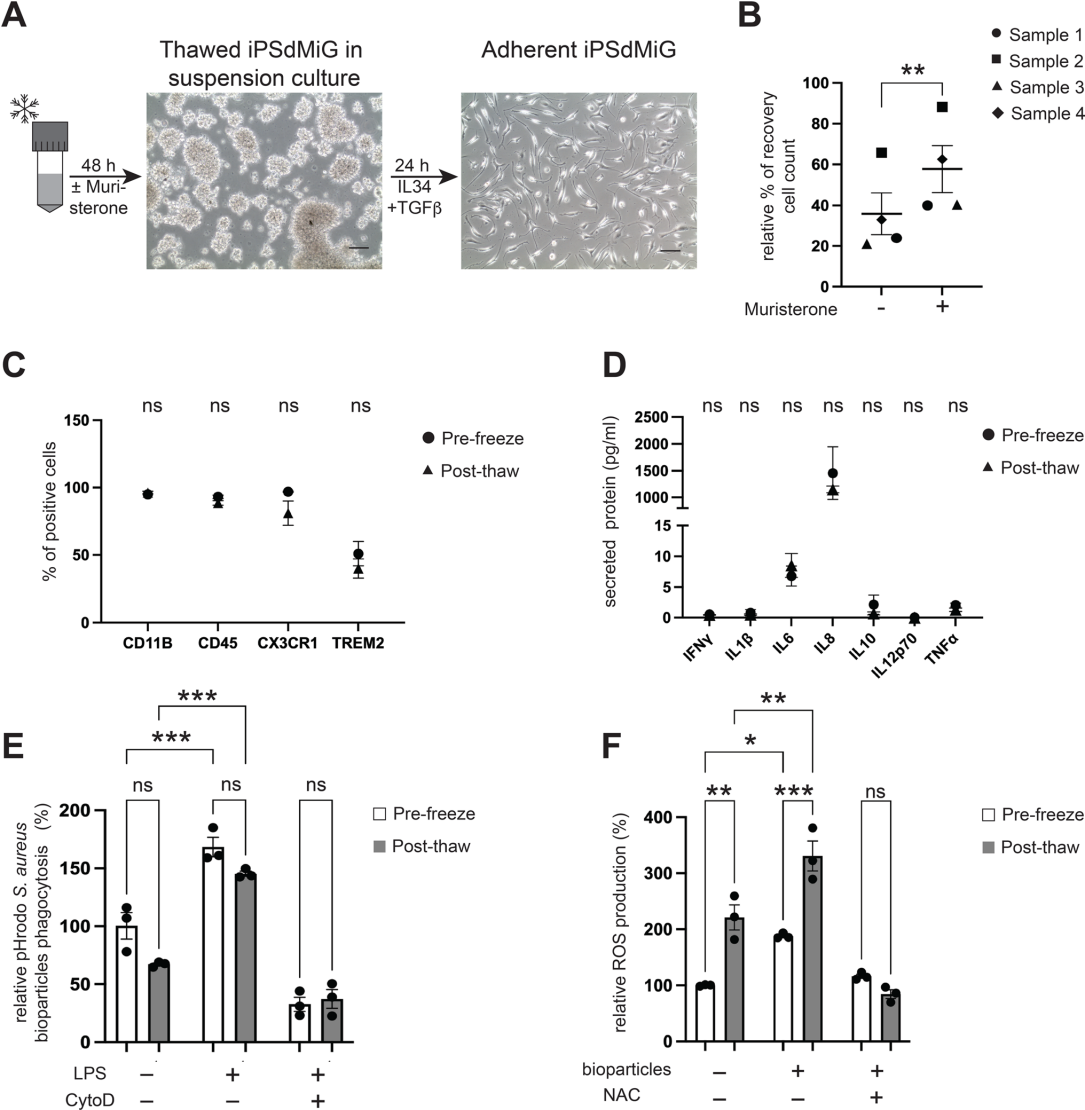
Efficient cryopreservation of iPSdMiG. **A** After thawing, iPSdMiG were maintained in suspension culture for 48 h, where they formed cell clusters, as was observed for suspension iPSdMiG before cryopreservation (compare Fig. 1B). Twenty-four hours after seeding, adherent iPSdMiG exhibit a ramified morphology, which is comparable to seeded iPSdMiG that were not cryopreserved (compare Fig. 1B). Scale bars = 100 μm. **B** The number of cells that recovered post-thawing was significantly increased by treatment with muristerone as compared to corresponding untreated samples. n = 4 paired independent experiments, mean ± SEM. ** p < 0.01 as determined by paired Students t-test. **C** The expression of the microglial surface markers CD11B, CD45, CX3C chemokine receptor 1 and TREM2 was comparable between pre-freeze and post-thaw iPSdMiG. n = 3 independent experiments, mean ± SEM. n.s = not significant as determined by unpaired two-way ANOVA followed by Bonferroni post-hoc test. **D** Concentrations of the secreted proteins IFNγ, IL1β, IL6, IL8, IL10, IL12p70 and TNFα were comparably low in pre-freeze and post-thaw iPSdMiG. n = 3 independent experiments, mean ± SEM. n.s = not significant as determined by unpaired two-way ANOVA followed by Bonferroni post-hoc test. **E** Phagocytosis of bioparticles in untreated, LPS-treated and CytoD-treated iPSdMiG was comparable before and after cryopreservation. Post-thaw iPSdMiG significantly increased phagocytosis after LPS stimulation compared to post-thaw unstimulated iPSdMiG. n = 3 independent experiments, mean ± SEM. n.s = not significant and * p < 0.05 as determined by unpaired two-way ANOVA followed by Bonferroni post-hoc test. **F** Constitutive and bioparticle-induced ROS levels before and after cryopreservation of iPSdMiG were significantly different, with cryopreserved cells exhibiting generally increased ROS levels. Nevertheless, iPSdMiG significantly induced ROS production after stimulation with bioparticles compared to untreated cells even after freeze-thawing. n = 3 independent experiments, mean ± SEM. n.s = not significant, * p < 0.05, ** p < 0.01 and *** p < 0.001 as determined by unpaired two-way ANOVA followed by Bonferroni post-hoc test

Since the production of iPSdMiG is a relatively long-lasting procedure spanning several months, the ability to cryopreserve iPSdMiG would greatly facilitate their use in downstream applications. In fact, difficulties to efficiently cryopreserve microglia have been a major limitation of previously published differentiation protocols. Using our protocol, we found that iPSdMiG (derived from Bioni010-C iPSCs) collected within the 6-week peak production phase are amenable to efficient cryopreservation using a standard freezing medium containing 90% KSR and 10% DMSO. For recovery, thawed iPSdMiG were first cultured in non-tissue culture suspension plates for 48 h (Fig. 5A). When seeded for adherent culture, they acquired their typical ramified morphology within 24 h (Fig. 5A). The recovery of frozen cells could be significantly enhanced by treatment with the anti-apoptotic molecule muristerone (5 μM; for 24 h prior to freezing as well as post-thawing). Addition of muristerone increased the recovery of viable cells after thawing from 33.0% ± 10.2% to 57.8% ± 11.4% (Fig. 5B).

Phenotypic and functional analyses of cryopreserved cells were performed after 3 to 12 months of storage at -150 °C. We found that under our optimized conditions, including treatment with muristerone, expression levels of the microglial-characteristic cell surface markers CD11B, CD45, CX3C chemokine receptor 1 and TREM2 remained stable after freeze-thawing (Fig. 5C and Suppl. Table S3). The same applied to the constitutive secretome profile of cryopreserved iPSdMiG (Fig. 5D and Suppl. Table S4). Furthermore, phagocytosis rates in response to bioparticle stimulation, under baseline conditions as well as upon treatment with LPS alone or in conjunction with CytoD, were comparable in iPSdMiG before and after cryopreservation. Most importantly, iPSdMiG were still capable of increasing their phagocytotic activity upon stimulation with LPS after thawing (Fig. 5E and Suppl. Table S5). However, the rates of constitutive and induced ROS production differed significantly between cryopreserved and non-cryopreserved cells. Nevertheless, a significant increase in ROS production was still detectable in bioparticle-stimulated cryopreserved iPSdMiG as compared to unstimulated controls (Fig. 5F and Suppl. Table S6). Altogether, these data demonstrate that cryopreservation of iPSdMiG altered neither their cellular and secretory phenotype, nor their phagocytic response to stimuli. Although ROS production was also still inducible upon bioparticle stimulation in iPSdMiG post cryopreservation, levels were overall elevated as compared to fresh microglia.

### IPSdMiG Readily Integrate Into Neural 2D and 3D Cultures

**Fig. 6:**
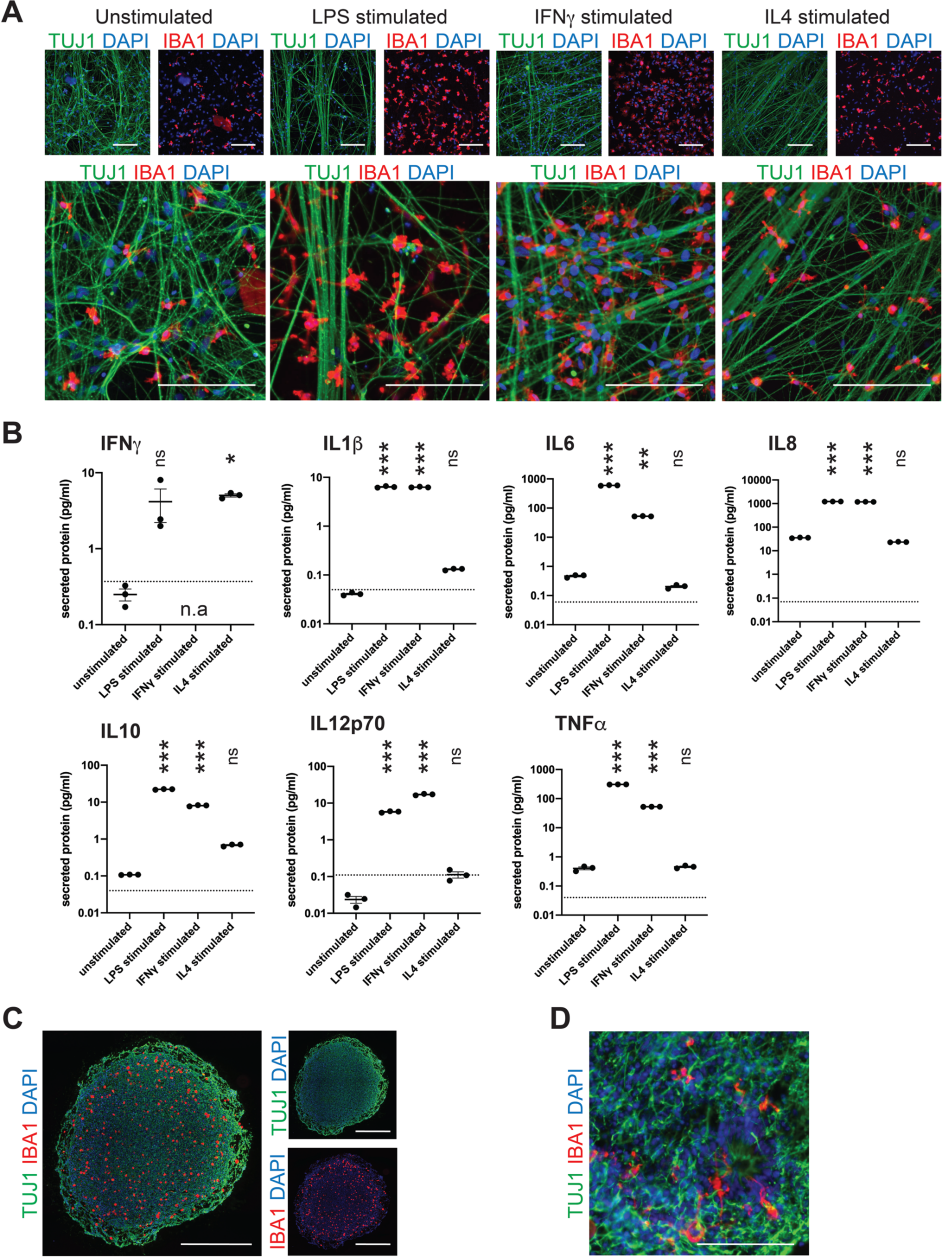
Integration of iPSdMiG into neural 2D and 3D cultures. **A** Exemplary immunofluorescence images of iPSdMiG (IBA1, red), integrating in lt-NES-derived neuronal networks (TUBB3, green) under unstimulated, LPS-, IFNγ- and IL4-stimulated conditions. Scale bars = 50 μm. **B** Secretion of the inflammatory cytokines and chemokines IL1β, IL6, IL8, IL10, IL12p70 and TNFα increased after stimulation with LPS or IFNγ. Except for IFNγ secretion, stimulation with IL4 had no effect on the secretome of microglial-neural co-cultures as compared to the secretory profile of unstimulated control cultures. n = 3 independent experiments, mean ± SEM. n.s. = not significant, * p < 0.05, ** p < 0.01, *** p < 0.001 as determined by one-way ANOVA followed by Bonferroni post hoc (IFNγ, IL1β, IL6, IL8, IL12p70 and TNFα) and Kruskal–Wallis test (IL10). n.a = not applicable; in reference to IFNγ measurement under IFNγ-treated conditions. Dotted line = lower detection limit. **C** Representative immunofluorescence images demonstrating that iPSdMiG (IBA1, red) migrated into and evenly distribute across 3D cortical spheroids (TUBB3, green); nuclei stained with DAPI. Scale bars = 500 μm. **D** High magnification images revealing that integrated iPSdMiG (IBA1, red) exhibit a ramified morphology after 4 weeks of co-culture with 3D cortical spheroids (TUBB3, green). Scale bar = 100 μm

To assess the amenability of iPSdMiG to 2D neural co-culturing, we harvested iPSdMiG from the medium of iLB-C133bm-S4 differentiation cultures, which exhibit a microglia surface marker expression pattern similar to that of adherent iPSdMiG monocultures (Suppl. Fig. S3A). Freshly harvested iPSdMiG were directly seeded onto adherent 2D cultures of lt-NES-derived neurons [46], which were pre-differentiated for 5 weeks and had thus already formed a dense neuronal network. Co-culture was carried out for 5 days in the presence of rhIL34. RhIL34 was then withdrawn from the culture medium, and cells were either stimulated with the pro-inflammatory stimuli LPS or IFNγ, or treated with the anti-inflammatory cytokine IL4 for 24 h before harvesting. Immunofluorescence staining of these 5-day-old co-cultures revealed that the IBA1-positive iPSdMiG had evenly distributed and integrated in the pre-formed TUBB3-positive neuronal network (Fig. 6A). Notably, treatment with the pro-inflammatory stimuli LPS and IFNγ, but not the anti-inflammatory stimulus IL4, significantly increased the levels of the inflammatory cytokines IL1β, IL6, IL8, IL10, IL12p70 and TNFα in the supernatant of these microglia-neuronal co-cultures (Fig. 6B and Suppl. Table S7). Interestingly, stimulation with IL4 induced a significant increase in IFNγ secretion compared to untreated co-cultures (Fig. 6B and Suppl. Table S7).

Finally, we addressed whether iPSdMiG also have the capacity to integrate into pre-formed 3D cortical spheroids. To this end, suspension iPSdMiG were added to 30-day-old cortical spheroid cultures generated according to a protocol adapted from Pasca and colleagues [47]. Specifically, ten cortical spheroids and 5 × 10^6^ iPSdMiG were added to the same CERO tube and placed in a CERO tabletop bioreactor, where intermittent dynamic agitation during the first 3 days of co-culture facilitated microglial adherence to and consequential infiltration of the cortical spheroids. At the time of iPSdMiG addition, spheroids measured around 1 mm in diameter and were highly homogeneous in size and morphology. They displayed a complex cytoarchitecture recapitulating several aspects of early corticogenesis such as the formation of ventricular zone-like neuroepithelial rosettes, and expressed a wide range of neural markers including the pan-neuronal marker TUBB3, the neural stem cell markers SOX2 and PAX6, as well as the forebrain marker forkhead box protein 1 (FOXG1; data not shown). After 3 days of intermittent dynamic agitation, the cultures were switched to continuous dynamic agitation for the remaining culture period. After 1 week of co-culture, IBA1-positive iPSdMiG had migrated into and evenly distributed across the cortical spheroids (Fig. 6C). After 4 weeks, ramified microglia were found integrated in the spheroids’ TUBB3-positive neuronal network (Fig. 6D).

These experiments demonstrate that iPSdMiG are highly suitable for 2D and 3D neural co-cultures, where they migrate and integrate into pre-existing neuronal networks. As their response to treatment with pro-inflammatory stimuli indicates, they maintain their functional properties in such a co-culture setting and might thus also be suited for more complex in vitro disease models comprising diverse cell populations.

## Discussion

Neuroinflammation plays a key role in several neurodegenerative diseases, and microglia, as the resident immune cells of the CNS, are one of the main contributors to this pathology (reviewed by [9, 10]). Several studies have associated microglial gene variants with an increased risk of developing diseases such as Alzheimer’s and Parkinson’s [11–13]. Furthermore, microglial activation has been shown to contribute to the aggravation of disease pathology and participate in neurodegeneration [14–17]. Therefore, microglial in vitro models represent valuable systems for understanding the role of microglia in disease pathology, as well as for studying druggable target pathways for future therapeutic interventions.

During recent years, several protocols have been described that aim at deriving microglial-like cells from iPSCs [26–31]. However, most of the current approaches primarily make use of small molecule cocktails that predominantly yield myeloid precursors (for reviews see [57, 58]) without explicit exposure to a neural environment during the microglia induction phase, which is an important component of in vivo microglial development [21, 51, 59]. To account for this, some of the published protocols employ a secondary ‘priming’ step during microglia maturation, for example, by co-culturing microglial cells with primary neurons or astrocytes or exposing them to neural cytokine cocktails (for an overview of previously published protocols and their key aspects, as well as selected downstream applications, see Suppl. Table S8) [26–31, 60].

Here, we have set up an approach that enables side-by-side development of hemogenic endothelial cells and neuroepithelial precursors, giving rise to a population of mature microglial progenitors at later stages of differentiation, which are finally being released as mature microglia into the cell culture medium. This concomitant multilineage differentiation was achieved by a stepwise induction of EBs using rhBMP4, rhFGF2, rhACT A and WNTC59. RhBMP4, rhFGF2 and rhACT A have been previously described as players in mesoderm formation [61, 62]. Short-term treatment with rhBMP4 for just 24 h was shown to promote mesoderm formation in human embryonic stem cells, and this process could be further increased in the presence of rhACT A and rhFGF2 [61]. Notably, while rhBMP4 signaling is important for mesoderm formation, it has also been identified as a tightly regulated and crucial mediator of neurogenesis (as reviewed by [63, 64]). In particular, rhBMP4 has been demonstrated to overcome the anti-differentiation effects of rhFGF2-containing medium that promotes the proliferation of neural stem cells [64]. Therefore, despite the subsequent addition of rhFGF2, the early exposure of the cultures to rhBMP4 was still considered to support the induction of neuroepithelial cells. At the same time, we expected that the stepwise addition of rhFGF2 and rhACT A alongside rhBMP4 would promote mesoderm induction. In addition, we implemented inhibition of WNT signaling towards the end of the EB stage. This step was motivated by the observation that stage-specific inhibition of WNT signaling is required to drive hemogenic endothelial precursors toward primitive hematopoiesis, whereas activation of WNT promotes the development of definitive hematopoietic progenitors [48]. This paradigm finally yielded iPSdMiG, which were released in the cell culture medium and acquired a ramified morphology within just 24 h after plating. Acquisition of a *bona fide* microglial phenotype is supported by the expression of a number of characteristic markers including CD11B, CD45, CX3C chemokine receptor 1, TREM2, P2Y12 receptor and TMEM119, constitutive and inducible expression of inflammatory cyto-/chemokines, as well as inducible phagocytosis and phagocytosis-associated ROS production.

A major drawback of a number of currently available protocols is their limited scalability, which imposes significant restrictions for the use of microglia, for example, in large-scale screening applications. Typically, parallel propagation of large-sized adherent culture formats is used to tackle this issue, although this approach is rather inefficient in terms of labor time and cost. In contrast, suspension culture-based production comes with better scalability and volume-to-surface ratios, thereby improving both cell yield and relative costs [65]. This motivated us to develop a protocol that combines EB suspension culture with subsequent differentiation of the formed EBs on free-floating macrocarriers. In addition to improving volume-to-surface ratios as such, our macrocarrier approach overcomes the limitation that a significant fraction of microglial cells generated in adherent 2D cultures typically settles down on the culture substrate and thus escapes harvesting [30]. In contrast, our approach maintains the microglia-producing cell population anchored to a free-floating macrocarrier, from where harvestable microglial cells are released into the cell culture medium, thereby enabling direct and efficient capture of iPSdMiG. Additional advantages of using macrocarriers in dynamic suspension culture are the potential for largely unrestricted three-dimensional expansion and improved exchange of gases and nutrients, which supports long-term cultures of 12 weeks with no or only limited emergence of necrotic spots.

A particular feature observed in our EB-based differentiation paradigm is the formation of cystic structures, which started appearing from week 6 of differentiation and grew into larger macroscopic structures over time. These structures are lined by CD31-positive cells and frequently located in close vicinity to areas of neural differentiation. It is conceivable that the close apposition of microglia-containing cysts and neural cells may to some extent mimic priming processes native microglial cells undergo upon CNS invasion. While the formation of cysts appears to not be an indispensable requirement for the generation of microglia, as can be seen from the lack of cyst formation in directed differentiation protocols [27–29, 31], EB-based protocols have commonly described the formation of such structures [26, 30, 66]. However, in contrast to published protocols resulting in the formation of both, dense neuralized and non-neuralized cystic EBs [26, 66], our protocol yields EBs containing hemogenic endothelial and neural cells at the same time.

To validate the robustness of our protocol, we applied the differentiation paradigm to four genetically distinct control iPSC lines. The resulting iPSdMiG were quality-controlled by assessing expression of the cell surface epitopes CD11B, CD45, CX3C chemokine receptor 1 and TREM2, and secretion of inflammatory cyto-/chemokines across a time span of 6 weeks. These systematic time course analyses confirmed consistency across several harvests and independent differentiation runs. The obtained iPSdMiG also responded to *S. aureus* bioparticles by inducing both phagocytosis and phagocytosis-associated ROS production. Importantly, an in silico transcriptomic comparison to primary human microglia identified a 90% overlap of the top 20 core signature TFs expressed in both cell types. Pathways that were identified as differentially expressed between iPSdMiG and primary human microglia only included non-immune-related pathways such as ATP synthesis by chemiosmotic coupling and eukaryotic translation initiation.

Notably, the medium-size differentiation format and the multiple-harvest-scheme used in this study yielded 5- to 45-fold more iPSdMiG than input iPSCs. A gradual increase in yield was observed over time, peaking at week 10 of differentiation, after which the release of harvestable iPSdMiG declined until the cultures were discontinued at week 12 (data not shown). While in this study dynamic culture of four macrocarriers was performed in a single non-adherent T75 flask, an adjustable number of macrocarriers can, in principle, be cultured in a variety of suspension culture-based bioreactor systems. Therefore, we expect that the number of cells produced per run can be significantly increased by using larger formats and/or bioreactor systems. The aspect of scalability becomes especially important in the context of establishing large, quality-controlled microglia cell banks for biomedical downstream applications.

Another key prerequisite for such an approach is the possibility to cryopreserve iPSdMiG at the end of each lengthy production cycle. However, cryostorage and cell retrieval has previously been challenging for human microglial preparations. Hence, most reported iPSdMiG protocols lack a cryopreservation step (Suppl. Table S8). In contrast, our iPSdMiG population lends itself to efficient cryopreservation, and treatment with muristerone, a Bcl-XL inducer and apoptosis inhibitor [67], further increases post-freeze–thaw recovery. Importantly, the cellular phenotype as well as the functional immune responses of iPSdMiG are largely maintained after cryopreservation, making our differentiation scheme compatible with future cell banking endeavors. Only basal and induced ROS release levels were increased after thawing, which could point to potential activation of stress response pathways as a result of the cryopreservation process [68].

Disease modeling studies increasingly require more complex co-culture systems to fully recapitulate pathologies associated with more than one cell type. Considering this requirement, we also introduced iPSdMiG into both, 2D and 3D cultures of lt-NES cell-derived neurons and cortical spheroids, respectively. In both culture systems, iPSdMiG readily migrated into the dense neural networks, where they acquired a ramified morphology that could be maintained for several weeks. Notably, immunogenic responses of microglia to the pro-inflammatory stimuli LPS and IFNγ, but not the anti-inflammatory stimulus IL4, were confirmed by the induced production of inflammatory cytokines and chemokines in 2D co-culture. While these experiments serve as proof-of-concepts for the applicability of our iPSdMiG in neuronal co-culture settings, further studies are required to examine, for example, the impact of activated microglia on co-cultured neurons and whether stimulation-induced proliferation of microglia contributes to the observed increase in cyto-/chemokine release.

Taken together, we have established a developmentally informed protocol recapitulating key aspects of microglial ontogeny and differentiation, which efficiently and reproducibly yields large numbers of ready-to-use iPSdMiG. These cells are very similar to primary human microglia on transcriptome level and respond adequately to immunogenic stimulation. The capacity to cryopreserve iPSdMiG without altering their core phenotype or functional responses will prospectively allow the generation of large cell banks, comprising microglia from different healthy and diseased donors for biomedical applications. Finally, our study shows that iPSdMiG can be readily co-cultured with other neural cells in defined 2D and 3D systems, which further increases the usefulness of these cells in the context of disease modeling, drug screening and other biomedical applications.

## Supplementary Information

Below is the link to the electronic supplementary material.

Supplementary file1 (PDF 60133 kb)

Supplementary file2 (DOCX 15.6 kb)

## Data Availability

The RNA sequencing data generated in the course of this study have been deposited in NCBI’s Gene Expression Omnibus (GEO) and are accessible through GEO series accession number GSE178846 (https://www.ncbi.nlm.nih.gov/geo/query/acc.cgi?acc=GSE178846). All other datasets are available from the corresponding author upon reasonable request.

## References

[1] Tremblay MĚ, Lowery RL, Majewska AK (2010). Microglial interactions with synapses are modulated by visual experience. PLoS Biology.

[2] Stevens B (2007). The classical complement cascade mediates CNS synapse elimination. Cell.

[3] Schafer DP (2012). Microglia sculpt postnatal neural circuits in an activity and complement-dependent manner. Neuron.

[4] Peri F, Nüsslein-Volhard C (2008). Live imaging of neuronal degradation by microglia reveals a role for v0-ATPase a1 in phagosomal fusion in vivo. Cell.

[5] Hagemeyer N (2017). Microglia contribute to normal myelinogenesis and to oligodendrocyte progenitor maintenance during adulthood. Acta Neuropathologica.

[6] Wlodarczyk A (2017). A novel microglial subset plays a key role in myelinogenesis in developing brain. The EMBO journal.

[7] Prinz M, Jung S, Priller J (2019). Microglia Biology: One Century of Evolving Concepts. Cell.

[8] Badimon A (2020). Negative feedback control of neuronal activity by microglia. Nature.

[9] Bachiller S (2018). Microglia in neurological diseases: A road map to brain-disease dependent-inflammatory response. Frontiers in Cellular Neuroscience.

[10] Heneka MT (2019). Microglia take centre stage in neurodegenerative disease. Nature Reviews Immunology.

[11] Villegas-Llerena C, Phillips A, Reitboeck PG, Hardy J, Pocock JM (2016). Microglial genes regulating neuroinflammation in the progression of Alzheimer’s disease. Current Opinion in Neurobiology.

[12] Naj AC (2011). Common variants in MS4A4/MS4A6E, CD2uAP, CD33, and EPHA1 are associated with late-onset Alzheimer’s disease. Nature genetics.

[13] Hollingworth P (2011). Common variants in ABCA7, MS4A6A/MS4A4E, EPHA1, CD33 and CD2AP are associated with Alzheimer’s disease. Nature genetics.

[14] Onuska KM (2020). The dual role of microglia in the progression of Alzheimer’s disease. Journal of Neuroscience.

[15] Bernardino L, Volonté C, Passani MB, Ferreira R (2020). Editorial: Dual role of microglia in health and disease: Pushing the balance towards repair. Frontiers in Cellular Neuroscience.

[16] Rangaraju S (2018). Identification and therapeutic modulation of a pro-inflammatory subset of disease-associated-microglia in Alzheimer’s disease. Molecular Neurodegeneration.

[17] Olah M (2020). Single cell RNA sequencing of human microglia uncovers a subset associated with Alzheimer’s disease. Nature Communications.

[18] Tao Y, Zhang SC (2016). Neural subtype specification from human pluripotent stem cells. Cell Stem Cell.

[19] Kriks S (2011). Dopamine neurons derived from human ES cells efficiently engraft in animal models of Parkinson’s disease. Nature.

[20] Kirkeby A (2012). Generation of regionally specified neural progenitors and functional neurons from human embryonic stem cells under defined conditions. Cell Reports.

[21] Ginhoux F (2010). Fate mapping analysis reveals that adult microglia derive from primitive macrophages. Science.

[22] Hoeffel G (2015). C-Myb+ erythro-myeloid progenitor-derived fetal monocytes give rise to adult tissue-resident macrophages. Immunity.

[23] Schulz C (2012). A lineage of myeloid cells independent of Myb and hematopoietic stem cells. Science.

[24] Kierdorf K (2013). Microglia emerge from erythromyeloid precursors via Pu.1-and Irf8-dependent pathways. Nature Neuroscience.

[25] Goldmann T (2016). Origin, fate and dynamics of macrophages at CNS interfaces. Nature Immunology.

[26] Muffat J (2016). Efficient derivation of microglia-like cells from human pluripotent stem cells. Nature Medicine.

[27] Pandya H (2017). Differentiation of human and murine induced pluripotent stem cells to microglia-like cells. Nature Neuroscience.

[28] Abud EM (2017). iPSC-derived human microglia-like cells to study neurological diseases. Neuron.

[29] Douvaras P (2017). Directed Differentiation of Human Pluripotent Stem Cells to Microglia. Stem Cell Reports.

[30] Haenseler W (2017). A highly efficient human pluripotent stem cell microglia model displays a neuronal-co-culture-specific expression profile and inflammatory response. Stem Cell Reports.

[31] Takata K (2017). Induced-pluripotent-stem-cell-derived primitive macrophages provide a platform for modeling tissue-resident macrophage differentiation and function. Immunity.

[32] Schliwa M (1982). Action of cytochalasin D on cytoskeletal networks. Journal of Cell Biology.

[33] Wißfeld J (2021). Deletion of Alzheimer’s disease-associated CD33 results in an inflammatory human microglia phenotype. Glia.

[34] Dobin A (2013). STAR: Ultrafast universal RNA-seq aligner. Bioinformatics.

[35] Liao Y, Smyth GK, Shi W (2014). featureCounts: An efficient general purpose program for assigning sequence reads to genomic features. Bioinformatics.

[36] Risso D, Ngai J, Speed TP, Dudoit S (2014). Normalization of RNA-seq data using factor analysis of control genes or samples. Nature Biotechnology.

[37] R Core Team (2020). R: *A language and environment for statistical computing*. R Foundation for Statistical Computing, Vienna, Austria. https://www.R-project.org/.

[38] R Studio Team (2020). RStudio Integrated Development Environment for R. *RStudio* http://www.rstudio.com/).

[39] Love MI, Huber W, Anders S (2014). Moderated estimation of fold change and dispersion for RNA-seq data with DESeq2. Genome Biology.

[40] Zhu A, Ibrahim JG, Love MI (2019). Heavy-tailed prior distributions for sequence count data: Removing the noise and preserving large differences. Bioinformatics.

[41] Keenan AB (2019). ChEA3: Transcription factor enrichment analysis by orthogonal omics integration. Nucleic Acids Research.

[42] Almende, B. V., Thieurmel, B., & Robert, T. (2021). Package visNetwork - *Network Visualization using vis.js Library*. https://rdrr.io/cran/visNetwork/.

[43] Yu G, Wang LG, Han Y, He QY (2012). ClusterProfiler: An R package for comparing biological themes among gene clusters. OMICS: A Journal of Integrative Biology.

[44] Yu G, He QY (2016). ReactomePA: An R/Bioconductor package for reactome pathway analysis and visualization. Molecular BioSystems.

[45] Langfelder P, Horvath S (2008). WGCNA: An R package for weighted correlation network analysis. BMC Bioinformatics.

[46] Koch P, Opitz T, Steinbeck JA, Ladewig J, Brüstle O (2009). A rosette-type, self-renewing human ES cell-derived neural stem cell with potential for in vitro instruction and synaptic integration. Proceedings of the National Academy of Sciences of the United States of America.

[47] Pasca AM (2015). Functional cortical neurons and astrocytes from human pluripotent stem cells in 3D culture. Nature Methods.

[48] Sturgeon CM, Ditadi A, Awong G, Kennedy M, Keller G (2014). Wnt signaling controls the specification of definitive and primitive hematopoiesis from human pluripotent stem cells. Nature biotechnology.

[49] Dupré-Crochet S, Erard M, Nüβe O (2013). ROS production in phagocytes: Why, when, and where?. Journal of Leukocyte Biology.

[50] Forman HJ, Torres M (2002). Reactive oxygen species and cell signaling: Respiratory burst in macrophage signaling. American Journal of Respiratory and Critical Care Medicine.

[51] Bennett FC (2018). A combination of ontogeny and CNS environment establishes microglial identity. Neuron.

[52] Galatro TF (2017). Transcriptomic analysis of purified human cortical microglia reveals age-associated changes. Nature Neuroscience.

[53] Gosselin D (2014). Environment drives selection and function of enhancers controlling tissue-specific macrophage identities. Cell.

[54] Bharathan SP (2017). Systematic evaluation of markers used for the identification of human induced pluripotent stem cells. Biology Open.

[55] Butovsky O (2014). Erratum: Identification of a unique TGF-β-dependent molecular and functional signature in microglia (Nature Neuroscience (2014) 17 (131–143)). Nature Neuroscience.

[56] Cahoy JD (2008). A transcriptome database for astrocytes, neurons, and oligodendrocytes: A new resource for understanding brain development and function. Journal of Neuroscience.

[57] Speicher AM, Wiendl H, Meuth SG, Pawlowski M (2019). Generating microglia from human pluripotent stem cells: Novel in vitro models for the study of neurodegeneration. Molecular Neurodegeneration.

[58] Timmerman R, Burm SM, Bajramovic JJ (2018). An overview of in vitro methods to study microglia. Frontiers in Cellular Neuroscience.

[59] Gosselin D (2017). An environment-dependent transcriptional network specifies human microglia identity. Science.

[60] Konttinen H (2019). PSEN1ΔE9, APPswe, and APOE4 Confer Disparate Phenotypes in Human iPSC-Derived Microglia. Stem Cell Reports.

[61] Zhang P (2008). Short-term BMP-4 treatment initiates mesoderm induction in human embryonic stem cells. Blood.

[62] Lee TJ (2013). Enhancement of osteogenic and chondrogenic differentiation of human embryonic stem cells by mesodermal lineage induction with BMP-4 and FGF2 treatment. Biochemical and Biophysical Research Communications.

[63] Cole AE, Murray SS, Xiao J (2016). Bone Morphogenetic Protein 4 Signalling in Neural Stem and Progenitor Cells during Development and after Injury. Stem Cells International.

[64] Moon BS, Yoon JY, Kim MY, Lee SH, Choi T, Choi KY (2009). Bone morphogenetic protein 4 stimulates neuronal differentiation of neuronal stem cells through the ERK pathway. Experimental and Molecular Medicine.

[65] Derakhti S, Safiabadi-Tali SH, Amoabediny G, Sheikhpour M (2019). Attachment and detachment strategies in microcarrier-based cell culture technology: A comprehensive review. Materials science & engineering C Materials for biological applications.

[66] Amos PJ (2017). Modulation of hematopoietic lineage specification impacts TREM2 expression in microglia-like cells derived from human stem cells. ASN Neuro.

[67] Oehme I, Bösser S, Zörnig M (2006). Agonists of an ecdysone-inducible mammalian expression system inhibit Fas Ligand- and TRAIL-induced apoptosis in the human colon carcinoma cell line RKO. Cell Death and Differentiation.

[68] Baust JM, Snyder KK, Van Buskirk RG, Baust JG (2022). Assessment of the impact of post-thaw stress pathway modulation on cell recovery following cryopreservation in a hematopoietic progenitor cell model. Cells.

