# Supplementary material for: Reenacting Neuroectodermal Exposure of Hematopoietic Progenitors Enables Scalable Production of Cryopreservable iPSC-Derived Human Microglia
Source: Stem Cell Rev Rep. 2022 Aug 15;19(2):455–74. doi: 10.1007/s12015-022-10433-w (PMC9902330; doi:10.1007/s12015-022-10433-w)
Supplement: Supplementary file 1 — Supplementary file1 (PDF 60133 kb) [file 12015_2022_10433_MOESM1_ESM.pdf]

**Supplementary Figure S1:** TRA-1-60 expression of iPSC lines used in this study

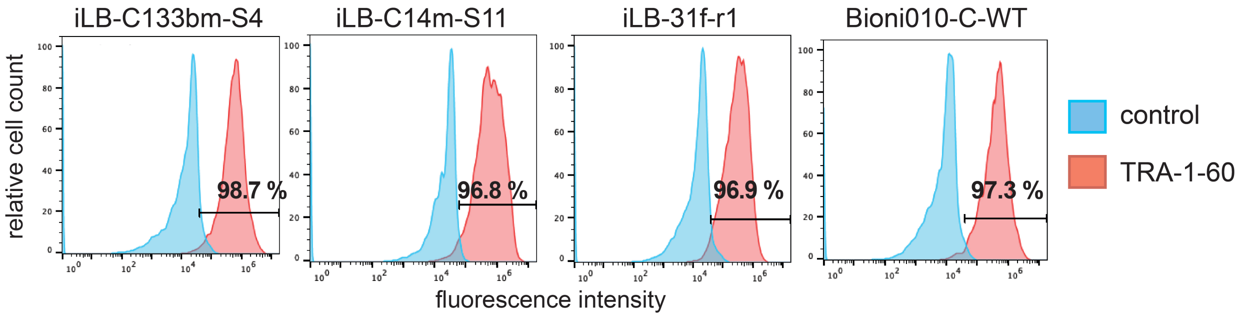


| | Sex | Reprogramming method | Age of donor |
|---|---|---|---|
| **iLB-C133bm-S4** | male | Sendai virus | 54 years |
| **iLB-C14m-S11** | male | Sendai virus | 55 years |
| **iLB-31f-r1** | female | retrovirus | 23 years |
| **Bioni 010 C** | male | episomal vector | 15 - 19 years |

**Supplementary Figure S2:** Macro- and microscopic visualization of cysts and differentiating tissue

**A** Photograph of cystic structures anchored on the macrocarrier

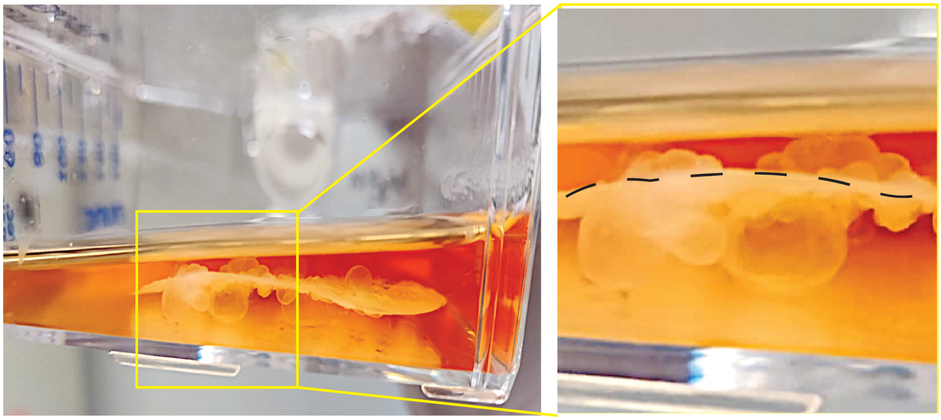

**B** Phase contrast image of a cyst containing round phase-bright microglia

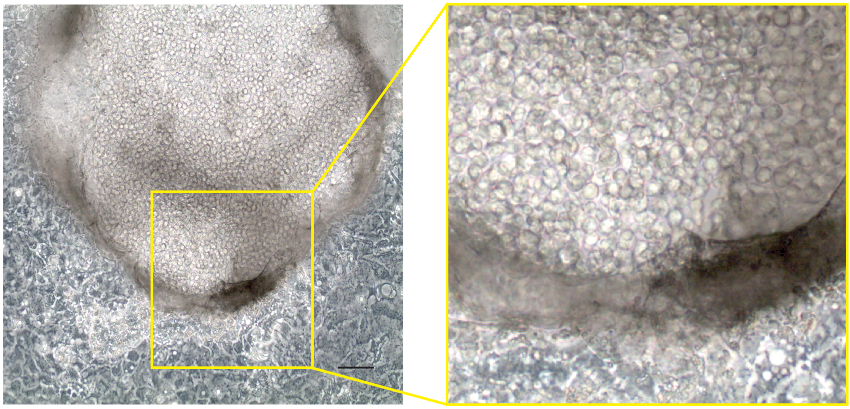

**C** Microscopy analysis of stained EB cryosections comprising cystic and condensed tissue-like structures

TUJ1 CD31 CD45 DAPI

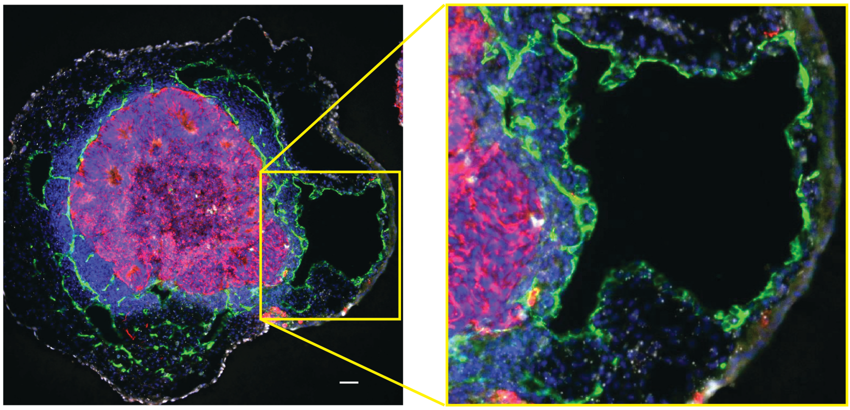

**Supplementary Figure S3:** Surface marker expression and proliferative index of iPSdMiG

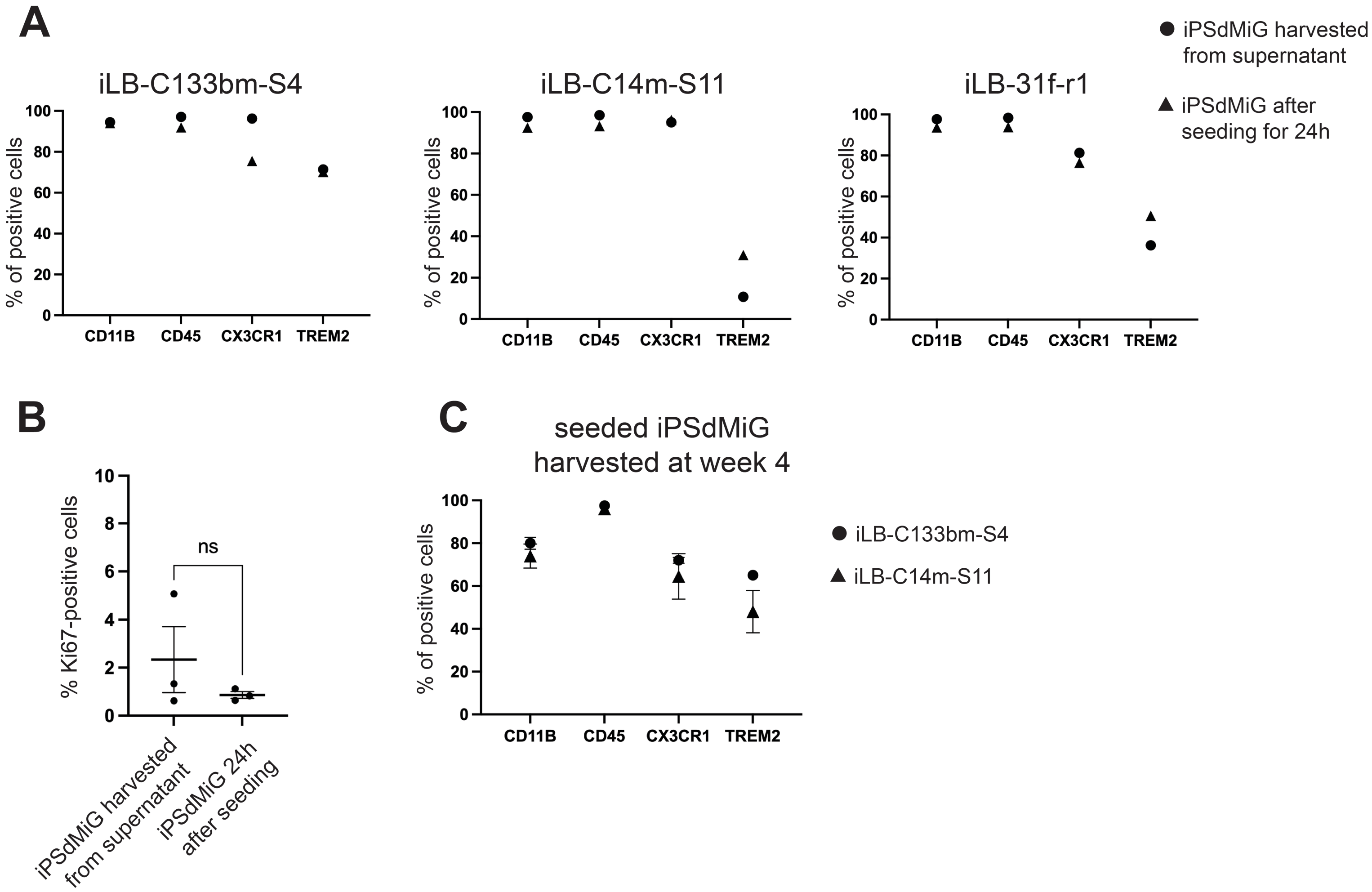

**Supplementary Figure S4:** RNA sequencing analysis of iPSdMiG in comparison to primary human microglia and iMGL microglia

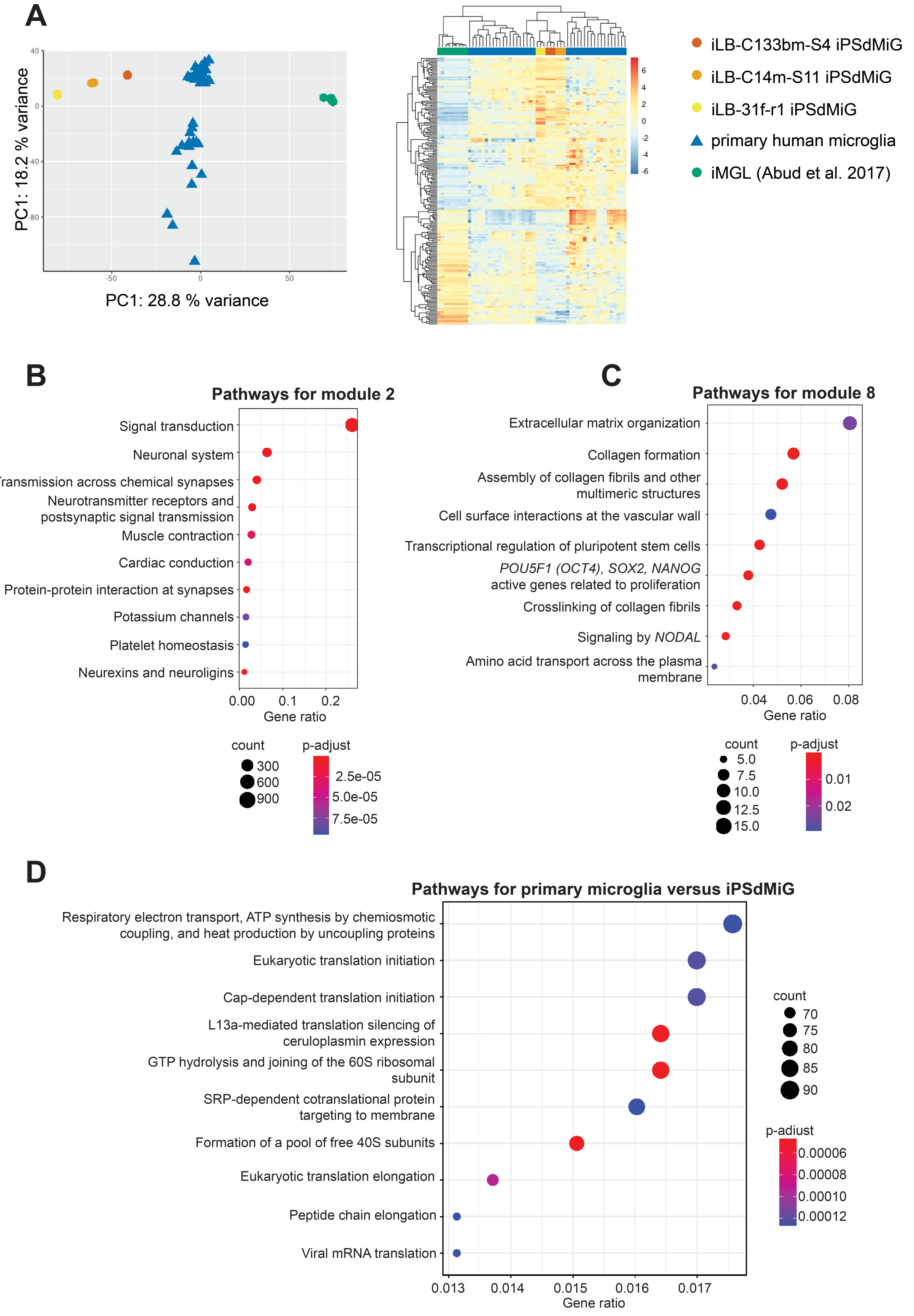

**Supplementary Table S1:** Microglial surface marker expression across different harvesting time points

| | Surface marker | harvest 1 W6 | harvest 2 W7 | harvest 3 W8 | harvest 5 W10 | harvest 7 W12 |
|---|---|---|---|---|---|---|
| iLB-C133bm-S4 (% of positive cells) | **CD11B** | 94 % ± 1 % | 93 % ± 2 % | 90 % ± 3 % | 93 % ± 2 % | 94 % ± 1 % |
| | **CD45** | 92 % ± 3 % | 95 % ± 3 % | 93 % ± 1 % | 89 % ± 3 % | 84 % ± 11 % |
| | **CX3CR1** | 76 % ± 4 % | 80 % ± 11 % | 92 % ± 4 % | 95 % ± 1 % | 97 % ± 0 % |
| | **TREM2** | 70 % ± 11 % | 59 % ± 6 % | 60 % ± 7 % | 49 % ± 4 % | 28 % ± 3 % |
| iLB-C14m-S11 (% of positive cells) | **CD11B** | 92 % ± 2 % | 94 % ± 2 % | 88 % ± 8 % | 95 % ± 2 % | 92 % ± 3 % |
| | **CD45** | 93 % ± 1 % | 93 % ± 2 % | 85 % ± 9 % | 90 % ± 4 % | 90 % ± 4 % |
| | **CX3CR1** | 96 % ± 1 % | 96 % ± 1 % | 89 % ± 8 % | 96 % ± 2 % | 94 % ± 3 % |
| | **TREM2** | 31 % ± 10 % | 43 % ± 17 % | 30 % ± 4 % | 21 % ± 10 % | 31 % ± 10 % |
| iLB-31f-r1 (% of positive cells) | **CD11B** | 94 % ± 2 % | 94 % ± 4 % | 91 % ± 5 % | 92 % ± 2 % | 93 % ± 3 % |
| | **CD45** | 94 % ± 3 % | 96 % ± 3 % | 89 % ± 3 % | 94 % ± 3 % | 90 % ± 3 % |
| | **CX3CR1** | 76 % ± 9 % | 93 % ± 1 % | 86 % ± 16 % | 98 % ± 0 % | 88 % ± 7 % |
| | **TREM2** | 51 % ± 4 % | 48 % ± 16 % | 42 % ± 2 % | 33 % ± 13 % | 44 % ± 9 % |

**Supplementary Table S2:** Constitutive secretion of inflammatory cytokines and chemokines across different harvesting time points

| | secreted protein (lower detection limit) | harvest 1 W6 | harvest 2 W7 | harvest 3 W8 | harvest 5 W10 | harvest 7 W12 |
|---|---|---|---|---|---|---|
| iLB-C133bm-S4 (pg/ml) | IFNγ (0.37) | 0.7 ± 0.2 | 0.2 ± 0.1 | 0.2 ± 0.1 | 0.2 ± 0.1 | 0.2 ± 0.0 |
| | IL1β (0.05) | 0.2 ± 0.1 | 0.3 ± 0.1 | 0.9 ± 0.7 | 0.1 ± 0.0 | 0.1 ± 0.1 |
| | IL6 (0.06) | 4.3 ± 1.3 | 1.9 ± 0.9 | 19.7 ± 15.2 | 1.0 ± 0.4 | 0.4 ± 0.1 |
| | IL8 (0.07) | 786.2 ± 387.7 | 670.6 ± 387.2 | 643.6 ± 59.2 | 605.7 ± 382.0 | 870.3 ± 167.3 |
| | IL10 (0.04) | 0.4 ± 0.3 | 0.4 ± 0.3 | 2.5 ± 0.1 | 0.2 ± 0.1 | 0.3 ± 0.1 |
| | IL12p70 (0.11) | 0.0 ± 0.0 | 0.0 ± 0.0 | 0.1 ± 0.0 | 0.0 ± 0.0 | 0.0 ± 0.0 |
| | TNFα (0.04) | 1.6 ± 0.3 | 1.1 ± 0.4 | 2.8 ± 0.8 | 1.0 ± 0.6 | 1.0 ± 0.2 |
| iLB-C14m-S11 (pg/ml) | IFNγ (0.37) | 0.4 ± 0.1 | 0.4 ± 0.3 | 0.2 ± 0.0 | 0.3 ± 0.1 | 0.2 ± 0.1 |
| | IL1β (0.05) | 0.5 ± 0.1 | 0.3 ± 0.2 | 0.3 ± 0.2 | 0.3 ± 0.2 | 0.3 ± 0.2 |
| | IL6 (0.06) | 24.5 ± 15.8 | 17.6 ± 17.1 | 17.9 ± 17.7 | 13.5 ± 13.3 | 13.6 ± 13.5 |
| | IL8 (0.07) | 1336.5 ± 1076.7 | 1183.9 ± 981.6 | 1153.0 ± 1006.2 | 944.2 ± 813.4 | 917.6 ± 806.3 |
| | IL10 (0.04) | 2.3 ± 0.9 | 1.5 ± 0.9 | 0.9 ± 0.5 | 0.9 ± 0.3 | 1.1 ± 0.4 |
| | IL12p70 (0.11) | 0.0 ± 0.0 | 0.0 ± 0.0 | 0.0 ± 0.0 | 0.0 ± 0.0 | 0.0 ± 0.0 |
| | TNFα (0.04) | 1.4 ± 0.6 | 1.1 ± 0.5 | 1.1 ± 0.6 | 1.1 ± 0.6 | 1.1 ± 0.5 |
| iLB-31f-r1 (pg/ml) | IFNγ (0.37) | 0.7 ± 0.1 | 0.4 ± 0.0 | 0.2 ± 0.0 | 0.2 ± 0.1 | 0.1 ± 0.0 |
| | IL1β (0.05) | 1.2 ± 0.6 | 1.1 ± 0.6 | 0.4 ± 0.2 | 0.3 ± 0.2 | 0.0 ± 0.0 |
| | IL6 (0.06) | 7.4 ± 1.8 | 5.3 ± 3.3 | 11.4 ± 5.8 | 10.0 ± 7.6 | 0.8 ± 0.2 |
| | IL8 (0.07) | 1448.9 ± 661.2 | 1447.6 ± 501.1 | 1901.7 ± 811.4 | 690.5 ± 220.9 | 958.4 ± 502.9 |
| | IL10 (0.04) | 2.9 ± 1.9 | 2.9 ± 2.0 | 2.7 ± 0.3 | 1.4 ± 0.9 | 0.7 ± 0.3 |
| | IL12p70 (0.11) | 0.1 ± 0.0 | 0.0 ± 0.0 | 0.0 ± 0.0 | 0.0 ± 0.0 | 0.0 ± 0.0 |
| | TNFα (0.04) | 2.0 ± 0.4 | 1.9 ± 0.5 | 1.9 ± 0.6 | 0.9 ± 0.1 | 1.2 ± 0.3 |

**Supplementary Table S3:** IPSdMiG surface marker expression before and after cryopreservation

| | Surface marker | Pre-freeze | Post-thaw |
|---|---|---|---|
| Bioni010 C WT (% of positive cells) | **CD11B** | 95 % ± 1 % | 96 % ± 1 % |
| | **CD45** | 93 % ± 1 % | 89 % ± 2 % |
| | **CX3CR1** | 97 % ± 1 % | 81 % ± 9 % |
| | **TREM2** | 51 % ± 9 % | 40 % ± 7 % |

**Supplementary Table S4:** Constitutively secreted inflammatory cytokines and chemokines before and after cryopreservation

| | secreted protein (lower detection limit) | Pre-freeze | Post-thaw |
|---|---|---|---|
| Bioni010 C WT (pg/ml) | **IFNγ (0.37)** | 0.7 ± 0.2 | 0.5 ± 0.1 |
| | **IL1β (0.05)** | 0.62 ± 0.5 | 0.7 ± 0.3 |
| | **IL6 (0.06)** | 5.6 ± 1.9 | 11.8 ± 6.7 |
| | **IL8 (0.07)** | 1154.4 ± 529.9 | 1780.9 ± 522.5 |
| | **IL10 (0.04)** | 1.6 ± 1.4 | 1.1 ± 1.1 |
| | **IL12p70 (0.11)** | 0.3 ± 0.0 | 0.1 ± 0.0 |
| | **TNFα (0.04)** | 1.9 ± 0.4 | 1.8 ± 0.4 |

**Supplementary Table S5:** Phagocytosis of bioparticles before and after cryopreservation

| Bioni010 C WT (pg/ml) | pre-freeze | post-thaw |
|---|---|---|
| **unstimulated** | 100 % ± 11 % | 67 % ± 1 % |
| **LPS stimulated** | 168 % ± 8 % | 145 % ± 2 % |
| **CytoD** | 33 % ± 6 % | 37 % ± 8 % |

**Supplementary Table S6:** Phagocytosis-associated ROS production before and after cryopreservation

| Bioni010 C WT (pg/ml) | pre-freeze | post-thaw |
|---|---|---|
| **unstimulated** | 100 % ± 1 % | 221 % ± 23 % |
| **bioparticles stimulated** | 172 % ± 9 % | 330 % ± 27 % |
| **NAC** | 116 % ± 4 % | 84 % ± 8 % |

**Supplementary Table S7:** Induced secretion of inflammatory cytokines and chemokines in 2D co-culture

| | secreted protein (lower detection limit) | Unstimu-lated | LPS stimulated | IFNγ stimulated | IL4 stimulated |
|---|---|---|---|---|---|
| co-cultured iPSdMiG and lt-NES-derived neurons® (pg/ml) | **IFNγ (0.37)** | 0.2 ± 0.0 | 4.2 ± 1.0 | n.a | 5.0 ± 0.0 |
| | **IL1β (0.05)** | 0.0 ± 0.0 | 6.4 ± 9.7 | 6.3 ± 0.5 | 0.1 ± 0.0 |
| | **IL6 (0.06)** | 0.5 ± 0.0 | 595.9 ± 9.7 | 52.2 ± 0.5 | 0.2 ± 0.0 |
| | **IL8 (0.07)** | 35.4 ± 0.8 | 1216.8 ± 1.9 | 1181.3 ± 5.3 | 23.4 ± 0.4 |
| | **IL10 (0.04)** | 0.1± 0.0 | 22.2 ± 0.3 | 8.0 ± 0.2 | 0.7 ± 0.0 |
| | **IL12p70 (0.11)** | 0.0 ± 0.7 | 5.8 ± 1.9 | 17.2 ± 5.3 | 0.1 ± 0.4 |
| | **TNFα (0.04)** | 0.4 ± 0.0 | 308.3 ± 0.9 | 52.7 ± 0.2 | 0.5 ± 0.0 |

**Supplementary Table S8:** Overview of published protocols for the generation of iPSC-derived microglial precursors / microglial-like cells

| | **Nomelcla-ture** | **Culture method & scale** | **Sorting /purifi-cation** | **Exposure to neuroec-todermal cells** | **Yield** | **Cryopreservation & recovery** | **Assessed applications by original publication and other groups** |
|---|---|---|---|---|---|---|---|
| **Muffat et al 2016** [26] | pMGLs | EB-based with fur-ther differentiation in tissue culture plates | Trituration of se-lected cystic EBs | Optional; direct or in-direct co-culture with neurons and astro-cytes | 0.5 - 4 fold of input iPSCs | Not described | 2D and 3D co-cultures with neurons and glia, and MECP2-mutant microglia [26]; Zika virus infection in de-veloping microglia [70]; APOE4 iMGL / clearing amyloid beta [71]. |
| **Abud et al 2017** [28] | iMGLs | 2D differentiation in 6-well tissue culture plates | FACS $CD43^+$ hematopoietic progenitors | Optional; 3 days of maturation with rat hippocampal neurons | 30 - 40 fold of input iPSCs | Not described | Invasion of iMGLs into 3D organoids [28]; TREM2-risk variant iMGLs [72]. |
| **Pandya et al 2017** [27] | iPS-MG | 2D differentiation on astrocytes in tissue culture plates | FACS $CD39^+$ microglia-like cells | Differentiation on as-trocytes | 0.8 - 3 fold of input iPSCs | Not described | Glioma-bearing mouse model [27]. |
| **Douvaras et al 2017** [29] | iPSC-MG | 2D differentiation in 6-well tissue culture plates | FACS $CD14^+$ $CX3CR1^+$ mi-croglial progeni-tors | Not described | 2 - 8 fold of input iPSCs | Post-thaw viability of progenitors is 57 % ± 5 % | TREM-risk variant iPSC-MG (modified protocol [73]). |
| **Haenseler et al 2017** [30] | pMGL / co-pMG | EB-based differentia-tion in up to T175 tissue culture flasks | Not required | 14-day co-culture of harvested pMacpre with cortical neurons to obtain mature co-pMG | 10 - 43 fold of input iPSCs | Not described | TREM2-risk variant pMGL [74]; [75]; [76] and [77]. |
| **Takata et al 2017** [31] | iMacs | 2D differentiation in 6-well tissue culture plates | FACS $CD45^+$ $CD11B^+$ $CD163^+$ $CD14^+$ $CX3CR1^+$ iMacs | 14-day co-culture of iMacs with iPSC-de-rived neurons to obtain mature iMicros | 10 - 20 fold from monoculture and 0.5 - 4 fold from EB-based differ-entiation of input iPSC | Not described | Transplantation into the postnatal mouse brain and in-tra-nasally into mouse lung. MEFV-mutant microglia [31]. |
| **Kontinnen et al 2019** [61] | iMGLs | 2D differentiation in tissue culture plates | Not required | Not described | 20 fold of input iPSCs | Not described | Co-culture in 3D matrix with iPSC-derived neurons and cerebral organoids [61]. |
| **This study** | iPSdMiG | EB-based differentia-tion on novel macro-carriers for scalable differentiation | Not required | Inherent exposure to neuroectodermal cells during differentiation; no additional matura-tion required | 5 - 45 fold of input iPSCs | Post-thaw viability of mature microglia is 57.8 % ± 11.4 % | Co-culture with 2D ltNES-neurons and 3D cortical spheroids. |

[28]
