# Supplementary material for: Reenacting Neuroectodermal Exposure of Hematopoietic Progenitors Enables Scalable Production of Cryopreservable iPSC-Derived Human Microglia
Source: Stem Cell Rev Rep. 2022 Aug 15;19(2):455–74. doi: 10.1007/s12015-022-10433-w (PMC9902330; doi:10.1007/s12015-022-10433-w)
Supplement: Supplementary file 2 — Supplementary file2 (DOCX 15.6 kb) [file 12015_2022_10433_MOESM2_ESM.docx]

**Supplementary Figures**

**Supplementary Figure S1. TRA-1-60 expression of iPSC lines used in this study.**

For all four cell lines used in this study, more than 96 % of all iPSCs expressed the pluripotency-associated marker TRA-1-60 as assessed by flow cytometry analysis (red; compared to isotype control antibody in blue). n = 1 per cell line. The iPSC lines iLB-C133bm-S4 and iLB-C14m-S11 were previously generated by reprogramming blood samples obtained from a 54-year-old and a 55-year-old male donor, respectively, using Sendai viruses. IPSC lines iLB-31f-r1 and Bioni010-C were previously obtained by reprogramming fibroblasts of a 23-year-old female donor using retroviruses and a 15- to 19-year-old male donor using episomal vectors, respectively.

**Supplementary Figure S2. Macro- and and microscopic visualization of cysts and differentiating tissue.**

(A) Photograph of cystic structures anchored to the free-floating macrocarrier membranes at week 12 of differentiation. Insert shows zoom view with dashed line indicating the macrocarrier. (B) Phase contrast image of a cyst containing round phase-bright microglia. Scale bar = 200 μm. (C) Immunofluorescence picture of a cryosectioned EB showing juxtapositioning of a cystic structure lined by CD31-positive cells and solid TUBB3-positive tissue. Note that the cysts’ contents typically vanished during cryosectioning and immunostaining procedures. Scale bar = 100 μm.

**Supplementary Figure S3. Surface marker expression and proliferative index of iPSdMiG.**

(A) Flow cytometry analysis of CD11B, CD45, CX3C chemokine receptor 1 and TREM2 expression. IPSdMiG directly harvested from the cell culture medium at week 6 of differentiation exhibited an expression profile comparable to adherent iPSdMiG. n = 1 per cell line. (B) For both, iPSdMiG directly harvested from the cell culture medium and adherent iPSdMiG cultures assessed 24 hours after seeding, 1 % - 2 % of the population expressed the proliferation marker Ki67 (assessed by flow cytometry; harvested after 6 weeks of differentiation). n = 3 independent experiments, mean ± SEM. n.s. = not significant as determined by Welch’s t-test. (C) Flow cytometry analysis of CD11B, CD45, CX3C chemokine receptor 1 and TREM2 expression in week 4 harvests collected from cell lines iLB-C133bm-S4 and iLB-C14m-S11. n = 2 independent experiments per cell line, mean ± standard deviation.

**Supplementary Figure S4. RNA sequencing analysis of iPSdMiG in comparison to primary human microglia and iMGLs.**

(A) Left: PCA corresponding to Fig. 4A but without iPSCs, THP1 macrophages and cortex samples. Right: Heat map representation of the top 200 DEGs in the the same microglial sample collection. Hierarchical clustering reveals close similarity of iPSdMiG and primary human microglia. (B) Module 2, which correlated with the cortex samples, showed enrichment for neuronal-related pathways. (C) Overrepresentation analysis of genes in module 8, which was highly correlated with iPSCs, revealed enrichment for pluripotency and extracellular matrix-related pathways. (D) Pathway enrichment analysis for genes differentially expressed in primary human microglia versus iPSdMiG (|log_2_FC| ≥ 1 and FDR-adjusted p-value ≤ 0.01) revealed differences in protein biosynthesis pathways. Notably, no immunity-related differences were identified.

**Supplementary Table S1.** Microglial surface marker expression across different harvesting time points.

**Supplementary Table S2.** Constitutive secretion of inflammatory cytokines and chemokines across different harvesting time points.

**Supplementary Table S3.** IPSdMiG surface marker expression before and after cryopreservation.

**Supplementary Table S4.** Constitutively secreted inflammatory cytokines and chemokines before and after cryopreservation.

**Supplementary Table S5.** Phagocytosis of bioparticles before and after cryopreservation.

**Supplementary Table S6.** Phagocytosis-associated ROS production before and after cryopreservation.

**Supplementary Table S7.** Induced secretion of inflammatory cytokines and chemokines in 2D co-culture.

**Supplementary Table S8.** Overview of published protocols for the generation of iPSC-derived microglial precursors / microglial-like cells. The table lists the proprietary nomenclature of the microglial cell populations, culture method and scale, sorting/purification steps, exposure to neuroectodermal cells, yield, cryopreservation and recovery, and selected applications reported by the original publication and other groups.

**SUPPLEMENTARY REFERENCES**

[69] J. Muffat *et al.*, (2018). Human induced pluripotent stem cell-derived glial cells and neural progenitors display divergent responses to Zika and dengue infections. Proceedings of the National Academy of Sciences of the United States of America, 115(27), 7117–7122.

[70] Y. T. Lin *et al.*, (2018). APOE4 causes widespread molecular and cellular alterations associated with Alzheimer’s disease phenotypes in human iPSC-derived brain cell types. Neuron, 98(6), 1141-1154.e7.

[71] J. Hasselmann *et al.*, (2019). Development of a chimeric model to study and manipulate human microglia in vivo. Neuron, 103(6), 1016-1033.e10.

[72] C. Claes *et al.*, (2019). Human stem cell–derived monocytes and microglia-like cells reveal impaired amyloid plaque clearance upon heterozygous or homozygous loss of TREM2. Alzheimer’s and Dementia, 15(3), 453–464.

[73] P. W. Brownjohn *et al.*, (2018). Functional studies of missense TREM2 mutations in human stem cell-derived microglia. Stem Cell Reports, 10(4), 1294–1307.

[74] P. Garcia-Reitboeck *et al.*, (2018). Human induced pluripotent stem cell-derived microglia-like cells harboring TREM2 missense mutations show specific deficits in phagocytosis. Cell Reports, 24(9), 2300–2311.

[75] X. Xiang *et al.*, (2018). The Trem2 R47H Alzheimer’s risk variant impairs splicing and reduces Trem2 mRNA and protein in mice but not in humans. Molecular Neurodegeneration, 13, 49.

[76] T. M. Piers *et al.*, (2020). A locked immunometabolic switch underlies TREM2 R47H loss of function in human iPSC-derived microglia. Federation of American Societies for Experimental Biology, 34(2), 2436–2450.
